# Nucleosome assembly protein-like 1 degradation-dependent novel cardioprotection mechanism of Wnt2 against ischemia‒reperfusion injury

**DOI:** 10.1038/s41392-025-02503-5

**Published:** 2025-12-16

**Authors:** Ying Wang, Liming Chen, Jinyi Lin, Xi Liu, Kejia Jin, Chenxing Huang, Hao Wang, Jianguo Jia, Jian Wu, Zhiwen Ding, Pan Gao, Junbo Ge, Hui Gong, Yunzeng Zou

**Affiliations:** 1 Shanghai Institute of Cardiovascular Diseases, State Key Laboratory of Cardiovascular Diseases, Zhongshan Hospital and Institutes of Biomedical Sciences, Fudan University, NHC Key Laboratory of Ischemic Heart Diseases, Key Laboratory of Viral Heart Diseases, Chinese Academy of Medical Sciences, Shanghai, China; 2 Institute of Advanced Medical Sciences and Huaihe Hospital, Henan University, Kaifeng, Henan Province China

**Keywords:** Cell biology, Cardiology

## Abstract

Wnt and its crosstalk signaling pathways are involved in the modulating ischemia‒reperfusion (I/R) injury. However, whether Wnt2 is a novel therapeutic agent for I/R injury is largely unknown. Here, we show that the downregulation of serum Wnt2 levels in acute myocardial infarction (AMI) patients following reperfusion therapy, and Wnt2 levels are inversely correlated with the levels of myocardial injury markers (cTnT and CK-MB). Therapeutic administration of recombinant Wnt2 protein (rbWnt2) alleviates cardiac I/R injury and improves cardiac function by suppressing ROS levels and cardiomyocyte death in mice. Further analysis revealed that rbWnt2 downregulated Nap1L1 to reactivate the transcription of antioxidant genes (SOD, GPX, and UCP3) to reduce ROS levels and subsequently inhibit cardiomyocyte apoptosis and ferroptosis during the I/R process. Cardiac-specific Nap1L1 knockdown attenuated I/R injury, whereas overexpression of Nap1L1 partly abolished the cardiac protection mediated by rbWnt2 administration in the I/R model. Mechanistically, Wnt2 promoted Nap1L1 ubiquitination and degradation to restore ROS scavenging systems via Lrp6-mediated recruitment of the E3 ligase Trim11 in I/R hearts. Nap1L1 suppression plays a critical role in mediating the cardioprotective effects of rbWnt2. These findings establish Wnt2 as a therapeutic agent that targets compartmentalized oxidative damage, suggesting a novel strategy to mitigate I/R injury through the Lrp6/Trim11/Nap1L1 axis.

## Introduction

Ischemic heart disease stands as a predominant contributor to global morbidity and mortality, representing a major and persistent challenge to public health systems worldwide.^1^ When ischemia occurs acutely, named acute myocardial infarction (AMI), which initiates a cascade of cellular events that can result in the irreversible death of cardiomyocytes and a precipitous decline in cardiac function. Timely myocardial reperfusion therapy is an effective strategy for restoring myocardial blood flow, rescuing viable cardiomyocytes and improving the prognosis of patients with AMI. Currently, thrombolytic or percutaneous coronary intervention (PCI) is the preferred emergency reperfusion strategy in most cases of AMI. However, reperfusion therapy paradoxically triggers additional cardiac damage, disorders and even death, which is termed ischemia‒reperfusion (I/R) injury. I/R injury remains a critical barrier to outcome improvement in acute myocardial infarction (AMI) patients undergoing revascularization therapies such as PCI.^2^ This process, which involves a complex interplay of metabolic, ionic, and inflammatory disturbances, significantly limits the full therapeutic benefit of revascularization. It is now well established that the initial minutes of reperfusion trigger rapid necrotic cell death, driven by opening of the mitochondrial permeability transition pore (mPTP) and inflammasome activation, which constitute the primary initiating event of infarction.^3,4^ This initial wave of destruction sets the stage for further damage. Building on this necrotic core, a secondary wave of regulated cell death unfolds over subsequent hours, substantially expanding infarct size and driving adverse remodeling. Among these delayed mechanisms, apoptosis (or mitochondrial Bax activation, cytochrome c release, etc.), necroptosis (a regulated inflammatory mode of cell death with features of apoptosis and necrosis) and ferroptosis (an iron dependent, lipid peroxidation-driven form of regulated programmed cell death) have emerged as critical executioners of cardiomyocyte loss during reperfusion.^5,6^ Currently, there is a lack of effective clinical interventions for cardiac I/R injury. Therefore, exploring feasible cardioprotective strategies for I/R injury is worthwhile for improving clinical outcome of patients with reperfusion therapy.

The search for such cardioprotective strategies has led researchers to investigate critical signaling pathways involved in I/R injury. Wnt proteins (Wnts), which are highly conserved in animals and widely expressed in various tissues, play important roles in the regulation of cell proliferation, differentiation, and death.^7^ Secreted Wnts serve as ligands to trigger the transduction of Wnt/β-catenin signaling to induce multiple biological functions by activating frizzled family receptors (FZDs) and low-density lipoprotein-receptor related protein (LRP) 5/6.^8,9^ Wnt signaling pathways, including the canonical Wnt/β-catenin, noncanonical Wnt/PCP and Wnt/Ca2+ signaling pathways, have become more critical for the modulation of organ I/R injury.^10^ Experimental evidences for their involvement in cardiac injury and protection are growing, recently. For instance, Wnt3a has been found to inhibit cardiomyocyte apoptosis by activating canonical Wnt/β-catenin signaling after myocardial I/R.^11^ Secreted SRP5 (sFRP-5) protects the heart from I/R injury by counteracting Wnt5a-mediated JNK signaling.^12^ Experimental MI increases the transcript levels of Wnt2, Wnt4, Wnt10b, and Wnt11 in heart tissue.^13^ Serum Wnt2 and Wnt4 levels are increased in AMI patients compared with healthy controls.^14^ Our preliminary experiments revealed that the serum level of Wnt2, but not that of Wnt4, is lower in AMI patients after PCI than in patients before PCI. Xia, S. et al. reported that Wnt2 overexpression protected against mitochondrial dysfunction and oxidative stress in Parkinson’s disease Drosophila model.^15^ Given the central role of mitochondrial dysfunction and oxidative stress in myocardial I/R injury, we speculate Wnt2 could protect against myocardial I/R injury by regulation of mitochondrial function. It needs further study to clarify.

Nucleosome assembly protein-like 1(Nap1L1), a member of the Nap protein family, plays an important role in the nuclear import of H2A-H2B and nucleosome assembly as a histone chaperone and participates in several important DNA repair mechanisms: it greatly enhances ERCC6-mediated chromatin remodeling, which is essential for transcription-coupled nucleotide excision DNA repair.^16^ This establishes its fundamental role in maintaining genomic integrity and regulating gene expression. In addition, we previously reported that Nap1L1 promoted dimethyl sulfoxide-induced differentiation of P19CL6 cells into cardiomyocytes,^17^ and knockdown of Nap1L1 induced mesoderm formation and cardiomyogenesis via notch signaling in murine-induced pluripotent stem cells.^18^ These findings point to a significant role for Nap1L1 in cardiac development and cell fate decisions. However, the roles and molecular mechanisms of Nap1L1 in cardiovascular diseases, especially in cardiac I/R injury, are largely unknown. In our preliminary study, we observed that Nap1L1 was increased during cardiac I/R injury, which was negatively correlated with decreased Wnt2 during the I/R procedure. The discovery of its inverse correlation with Wnt2 presents a novel axis in I/R injury, which is very worth for exploring.

Here, we identified Wnt2 as a dynamically downregulated factor in both clinical AMI cohorts and experimental I/R mouse models and revealed an inverse correlation with myocardial injury severity. Using multiomics and genetic approaches, we demonstrated that recombinant Wnt2 (rbWnt2) confers robust cardioprotection by suppressing ROS-triggered apoptosis and ferroptosis through a novel Lrp6/Trim11/Nap1L1 axis. This axis orchestrates the ubiquitin-mediated degradation of Nap1L1, relieving its repression of antioxidant response element (ARE)-dependent transcription and restoring redox homeostasis. Our findings reveal that Wnt2 is a master regulator of compartmentalized oxidative stress, suggesting a transformative therapeutic strategy to address the unmet need for precision interventions in ischemic heart disease.

## Results

### Wnt2 is negatively associated with cardiac I/R injury in AMI patients

**Fig. 1:**
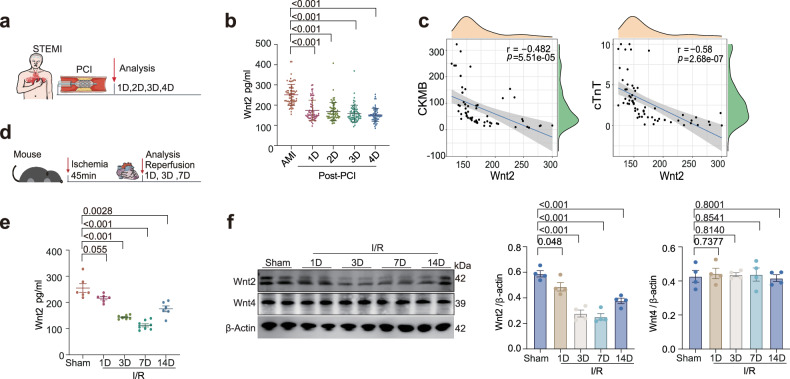
Decreased Wnt2 levels correlate with increased cardiac I/R injury in AMI patients. **a** Schematic diagram of the experimental workflow: Serum samples were collected from acute ST-segment elevation myocardial infarction (STEMI) patients at multiple time points before and after PCI. **b** Serum Wnt2 levels in STEMI patients before (AMI) and after PCI (Post-PCI). The data are presented as the means ± SDs (*n* = 68. **c** Scatter plots illustrating the relationships of serum Wnt2 levels (pg/mL) with cTNT(ng/mL) and CK-MB(U/L) in AMI patients during the first 48 h following PCI. Blood samples were collected at consistent time points across all patients. Correlations were evaluated via Pearson’s method; reported values include r values, two-sided *P* values, and 95% CIs (*n* = 68). **d** Graphical scheme for experimental workflows: Adult mice subjected to the I/R injury model; tissues from injured areas and serum were collected at designated reperfusion time points. **e** Serum Wnt2 levels were measured by ELISA in I/R-treated mice at different time points (*n* = 6‒8/group). **f** Relative Wnt2 and Wnt4 expression in the ischemic area of cardiac tissues from sham or I/R-treated mice (*n* = 4/group). The data are expressed as the means ± SEMs

To determine the clinical relevance of Wnt2 in cardiac I/R injury, we evaluated dynamic changes in serum Wnt2 levels in patients with acute myocardial infarction (AMI) before and after percutaneous coronary intervention (PCI) (Fig. 1a). Sixty-eight patients with acute ST-segment-elevation MI (STEMI) who received PCI were enrolled (Supplementary Table 1). Compared with those before reperfusion, post-PCI serum Wnt2 levels progressively decreased from days 1–4 (Fig. 1b). Cardiac troponin T (cTnT) and creatine kinase-MB (CK-MB) are both important biomarkers of myocardial injury. Elevated levels of these two markers are strongly correlated with the degree of myocardial injury and subsequent adverse events in MI patients.^19^ We found that the serum Wnt2 level was negatively correlated with the cTnT and CK-MB levels (Fig. 1c). The serum cTnT, CK-MB and Wnt2 levels were measured at the same time points at 48 h post-PCI.

These data revealed that reduced Wnt2 levels are associated with myocardial I/R injury, suggesting its potential role in protecting against I/R in the clinical setting.

### The administration of rbWnt2 successfully ameliorates cardiac I/R injury

To explore the pivotal role of Wnt2 in myocardial I/R injury, we systematically monitored serum and cardiac Wnt2 expression patterns in mice following I/R (Fig. 1d). Compared with those of the sham controls, the results of the quantitative ELISA analysis revealed a progressive decrease in the serum Wnt2 level from postoperative day 1 to day 7, and the level of Wnt2 began to rebound at 14 days of reperfusion (Fig. 1e). Cardiac Wnt2 levels showed similar expression profiles, whereas Wnt4 expression did not change at any time point post-I/R (Fig. 1f). Further analysis revealed that Wnt2 reduction was localized specifically to the ischemic area, with no significant change in remote regions compared with that in the sham group (Supplementary Fig. 1c). To specifically assess Wnt2 expression in cardiomyocytes at the early phase following I/R injury, we isolated cardiomyocytes from mice subjected to 45 min of ischemia followed by 1 h of reperfusion or a sham operation and observed that the Wnt2 level was markedly lower in cardiomyocytes at 1 h post-I/R injury than in those in the sham group (Supplementary Fig. 1b). In vitro, we isolated and cultured adult murine cardiomyocytes (AMCMs) subjected to hypoxia for 1 h followed by normoxia 1–6 h (H/N) to imitate I/R injury in vivo (Supplementary Fig. 1d). Compared with that in the control group, Wnt2 expression in AMCMs was significantly lower at 1 h, 3 h, and 6 h following H/N (Supplementary Fig. 1e). A reduction in Wnt2 was also observed in the CM collected from H/N-treated AMCMs relative to that collected from normoxia-treated cells (Supplementary Fig. 1f). Similarly, H/N stimulation led to decreased expression and secretion of Wnt2 in cultured cardiac fibroblasts (Supplementary Fig. 1g).

**Fig. 2:**
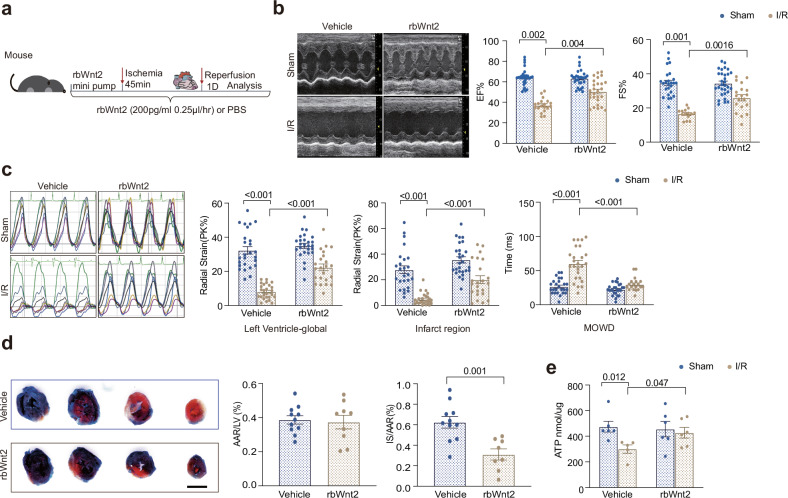
The administration of rbWnt2 significantly mitigates cardiac I/R injury. **a** Experimental design: Sham or I/R mice received chronic minipump infusion of PBS or recombinant human Wnt2 (rbWnt2, 200 pg/mL at 0.25 μL/h). **b** Representative M-mode echocardiograms and quantitative analysis of the left ventricular ejection fraction (LVEF) and fractional shortening (LVFS) at 24 h post-I/R. The mice were pretreated with rbWnt2 (200 pg/mL, 0.25 μL/h) or PBS (*n* = 14–24/group). **c** Radial strain analysis: Left: representative radial strain curves (colored lines indicate six myocardial segments). Right: Quantitative analysis of global/infarct zone radial strain and maximal opposite wall delay (MOWD), calculated as the temporal difference between peak segmental TPK values (ms) reflecting cardiac dyssynchrony (*n* = 14–27/group). **d** Evans blue/TTC staining of myocardial infarct areas (quantification shown; *n* = 8–11/group). IS/AAR: Infarct size/area at risk (% of AAR). Scale bar: 3 mm. **e** ATP levels in ischemic heart tissues from (**b**) (*n* = 6/group). The data are expressed as the means ± SEMs

To investigate the therapeutic potential of Wnt2 in myocardial I/R injury, we infused recombinant human Wnt2 (rbWnt2) into sham and I/R mice through continuous minipump infusion (200 pg/mL at 0.25 μL/h). Functional assessment and morphological detection were performed at 24 h post-I/R (Fig. 2a). Wnt2 infusion increased the level of Wnt2 in the heart tissue of both the sham and I/R groups (Supplementary Fig. 2a). Compared with the PBS (control) group, the rbWnt2 group presented profoundly improved cardiac function, as evidenced by the increased LVEF and LVFS in the I/R model (Fig. 2b). Wnt2 attenuated the increase in LVID but had minimal effects on left ventricular anterior wall thickness (LVAW) and heart rate (HR) (Supplementary Table 2). Furthermore, Wnt2 ameliorated diastolic dysfunction, as demonstrated by an improved E/A ratio in I/R model mice (Supplementary Fig. 2b). Advanced strain and strain rate analysis revealed that, compared with the control, rbWnt2 increased peak systolic strain (PK%, defined as the highest point achieved for the segment) in both the global ventricular and infarct zones. Additionally, the maximum opposite wall delay (MOWD), a parameter quantifying mechanical dyssynchrony on the basis of the temporal disparity in peak segmental contraction, was significantly reduced in rbWnt2-treated mice (Fig. 2c). Consistent improvements were observed in longitudinal strain, corroborating the radial strain findings (Supplementary Fig. 2c).

Morphometric assessment through Evans blue/TTC dual staining confirmed that rbWnt2 pretreatment significantly attenuated the myocardial infarction area (Fig. 2d). Adenosine triphosphate (ATP) is the source of energy for muscle contraction and is essential for maintaining cell integrity and survival and is sensitive to injury conditions.^20^ Notably, rbWnt2 administration preserved cardiac ATP levels post-I/R, a critical energy substrate for myocardial viability. No intergroup differences were detected in the sham controls (Fig. 2e). Additionally, we investigated the two-week effect of Wnt2 on myocardial I/R injury. The results indicated that cardiac function was restored in the I/R group and that Wnt2 did not further improve the LVEF (Supplementary Fig. 2e). We also conducted a series of in vitro experiments using cultured adult cardiomyocytes subjected to hypoxia/normoxia (H/N) with or without rbWnt2 treatment. RbWnt2 treatment effectively reversed the H/N-induced decrease in endogenous Wnt2 and ATP levels (Supplementary Fig. 2f–h), suggesting that Wnt2 has cardioprotective effects following I/R.

These data indicated that supplementation with exogenous rbWnt2 at the early phase post-I/R could confer cardioprotection against myocardial injury.

### RbWnt2 improves cardiomyocyte death during myocardial I/R injury

**Fig. 3:**
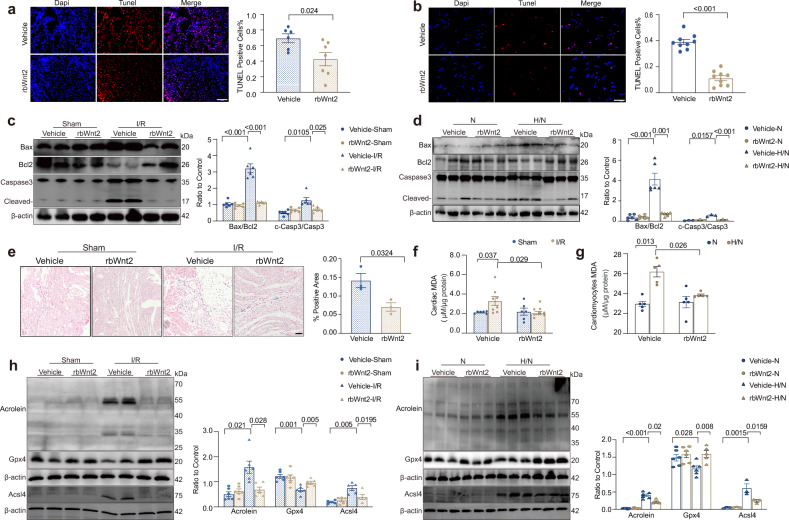
RbWnt2 intervention inhibits apoptosis and ferroptosis in myocardial I/R injury. **a** TUNEL staining analysis of I/R hearts from mice pretreated with rbWnt2 or PBS. Scale bar: 100 μm. *n* = 6–7/group. **b** TUNEL staining analysis of cultured AMCMs treated with rbWnt2 or PBS under 1 h of hypoxia/1 h of normoxia. Scale bar: 50 μm. *n* = 9/group. **c** Immunoblot analysis of the apoptosis-related proteins Bax, Bcl-2, cleaved caspase-3, and caspase- 3 in I/R or sham hearts from mice pretreated with rbWnt2 or PBS. *n* = 6/group. **d** Immunoblot analysis of the levels of the apoptosis-related proteins Bax, Bcl-2, cleaved caspase-3, and caspase-3 in cultured adult mouse cardiomyocytes (AMCMs) subjected to H/N or normoxia (N) and treated with rbWnt2 or PBS. *n* = 6/group. **e** Representative images of Prussian blue staining in the I/R injury area. Scale bar: 100 μm. *n* = 3/group. **f** Analysis of MDA levels in I/R or sham hearts from mice pretreated with PBS or rbWnt2. *n* = 6–9/group. **g** Malondialdehyde (MDA) levels in AMCMs subjected to H/N with rbWnt2 or PBS. *n* = 5/group. **h** Western blot of Acrolein, Gpx4 and Acsl4 in hearts from the groups described in (**c**). *n* = 5–6/group. **i** Western blot analysis of Acrolein, Gpx4 and Acsl4 expression in AMCMs under H/N conditions with rbWnt2 or PBS. *n* = 6/group. All the data are expressed as the means ± SEMs

Cardiomyocyte death involving necrosis, apoptosis, and ferroptosis is critical for halting both acute and progressive cardiac deterioration after I/R. TUNEL staining revealed that, compared with control hearts, rbWnt2-treated hearts presented significantly fewer TUNEL-positive nuclei post-I/R, indicating that Wnt2 inhibited cardiac apoptosis post-I/R (Fig. 3a). Consistently, rbWnt2 suppressed the I/R-induced activation of proapoptotic markers (cleaved caspase-3, caspase-3, Bax) while restoring antiapoptotic Bcl-2 expression (Fig. 3c). Parallel in vitro experiments confirmed that rbWnt2 inhibited H/N-triggered apoptosis via the intrinsic pathway in AMCMs (Fig. 3b, d). We also detected the expression of the extrinsic apoptosis marker caspase 8 and found that Wnt2 had no effect on the activation of caspase 8 induced by I/R (Supplementary Fig. 3a). These data demonstrated that Wnt2 mainly inhibited intrinsic apoptosis induced by I/R. Several studies have recently indicated the direct involvement of ferroptosis in I/R-induced oxidative stress damage.^21–23^ To confirm the effect of rbWnt2 on cardiomyocyte ferroptosis in particular, we treated cultured AMCMs with the ferroptosis inducers RSL3 and erastin. RbWnt2 pretreatment strongly alleviated the decrease in cardiomyocyte viability induced by RSL3 or Erastin (Supplementary Fig. 3b, c), suggesting that Wnt2 inhibits ferroptosis in cardiomyocytes following I/R. Ferroptosis is a type of programmed cell death induced by iron-dependent lipid peroxidation. In the I/R model, Prussian blue staining revealed that, compared with vehicle, rbWnt2 significantly reduced cardiac nonheme iron deposition (Fig. 3e). Acrolein, Acsl4 (acyl-CoA synthetase long-chain family member 4) and malondialdehyde (MDA) levels, which represent lipid peroxidation, were significantly increased in I/R-injured hearts compared with those in the sham group, and rbWnt2 greatly attenuated these effects, indicating that rbWnt2 reduced lipid peroxidation post-I/R (Fig. 3f, h). Gpx4 is an endogenous antioxidant of selenium-dependent enzymes that scavenges lipid peroxides and is a core regulator of the ferroptosis signaling pathway.^21,24^ As shown in Fig. 3h, I/R reduced Gpx4 expression, which was restored by rbWnt2 treatment. Similar effects were observed in H/N-injured AMCMs, where rbWnt2 reduced acrolein, Acsl4 and MDA levels and preserved Gpx4 expression (Fig. 3g, i). In addition, rbWnt2 mildly inhibited the release of LDH from cardiomyocytes after H/N, suggesting that rbWnt2 might improve necrosis post-I/R (Supplementary Fig. 3e). However, rbWnt2 had no inhibitory effect on the increased phosphorylation of Ripk1/Ripk3 or GSDMD-NT fragmentation post-I/R, indicating that rbWnt2 did not affect necrosis or pyroptosis induced by I/R (Supplementary Fig. 3d).

In summary, our findings demonstrate that rbWnt2 mitigates myocardial I/R injury by targeting cardiomyocyte death, including apoptosis and ferroptosis. These cardioprotective mechanisms underscore the therapeutic potential of rbWnt2 in preserving cardiomyocyte viability and attenuating both acute and chronic cardiac damage.

### Wnt2 reduces ROS levels by activating the transcription of antioxidant genes in I/R hearts

**Fig. 4:**
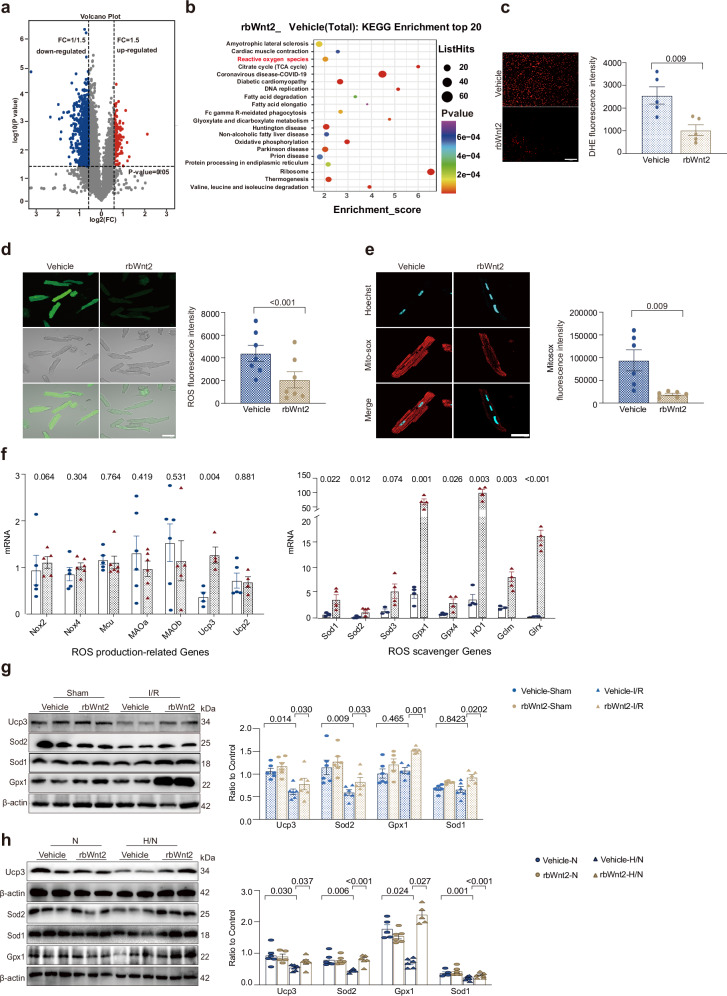
rbWnt2 enhances the transcription and expression of ROS-scavenging genes to mitigate oxidative stress. **a** Volcano plot of differentially expressed proteins in I/R-injured heart tissues from mice pretreated with rbWnt2 or PBS. **b** Top 20 KEGG pathways enriched with differentially expressed proteins. **c** Representative DHE-stained images of I/R hearts from the groups in (**a**). Scale bar: 100 μm. *n* = 5/group. **d** DCFH-DA staining of adult mouse cardiomyocytes (AMCMs) treated with rbWnt2 or PBS and subjected to H/N. Scale bar: 50 μm. *n* = 7/group. **e** MitoSOX staining of AMCMs subjected to H/N. Scale bar: 50 μm. *n* = 6/group. **f** Expression levels of ROS production-related genes (left panel, excluding Ucp3 and Ucp2) and ROS scavenger genes (right panel) in heart tissues from mice treated with PBS (vehicle) or rbWnt2 following ischemia/reperfusion (I/R) injury. *n* = 4–5/group. **g** Western blot analysis of Ucp3, Sod2, Sod1, and Gpx1 expression in sham or I/R hearts pretreated with PBS or rbWnt2. *n* = 6/group. **h** Western blot analysis of Ucp3, Sod2, Sod1 and Gpx1 expression in AMCMs pretreated with PBS or rbWnt2 after H/N. *n* = 5–6/group. All the data are expressed as the means ± SEMs

Next, we explored the underlying molecular mechanisms by which rbWnt2 protects against heart I/R injury. We performed proteomics analysis of hearts from I/R mice treated with rbWnt2 or vehicle. The data revealed 170 upregulated proteins and 726 downregulated proteins in rbWnt2-treated hearts compared with vehicle-treated hearts (|log2FoldChange| > 1.5, false discovery rate (adjusted p value) <0.05) (Fig. 4a). KEGG pathway enrichment of the differentially expressed proteins (DEPs) highlighted key pathways, including cardiac muscle contraction, reactive oxygen species (ROS) metabolism, the citrate cycle (TCA cycle), and oxidative phosphorylation (Fig. 4b). Given the established role of oxidative stress in I/R injury, where reperfusion triggers a pathological ROS burst, this burst intersects with metabolic, inflammatory, and cell death pathways to amplify tissue injury.^25,26^ Therefore, we evaluated ROS levels via DHE staining in vivo and DCFH-DA staining in vitro. RbWnt2 treatment significantly attenuated ROS accumulation in I/R-damaged cardiac tissues (Fig. 4c). A similar tendency was also observed in AMCMs stimulated with H/N (Fig. 4d). Mitochondria are the main source of cellular ROS.^27^ Mito-Sox staining revealed that the mitochondrial ROS level was lower in the rbWnt2-treated AMCMs than in the vehicle-treated AMCMs following H/N (Fig. 4e).

In mammalian cardiomyocytes, ROS homeostasis is dynamically maintained through coordinated production (via the ETC, MAO, NOX, and MCU) and elimination systems (primarily the SOD/GPX families).^28–30^ There was no significant difference in the mRNA levels of MAO, ETC, MCU and NOX in I/R hearts between the Wnt2 group and the control group. However, Wnt2 treatment significantly increased the transcription of the ROS-scavenging genes Sod1, Sod2, Sod3, Gpx1, Gpx4, HO1, Gclm, Glpx and Ucp3 (Fig. 4f). We then verified the expression of these proteins. RbWnt2 restored I/R-induced depletion of Ucp3, Sod1, Sod2 and Gpx1 in vivo (Fig. 4g), a parallel rescue observed in H/N-challenged AMCMs in vitro (Fig. 4h).

These findings reveal that rbWnt2 enhances the transcription and expression of key ROS-scavenging genes (SODs, GPXs, and UCP3) involved in mitigating oxidative stress and restoring redox homeostasis in cardiomyocytes following I/R.

### Wnt2 downregulates Nap1L1 to reactivate antioxidant gene transcription in I/R hearts

**Fig. 5:**
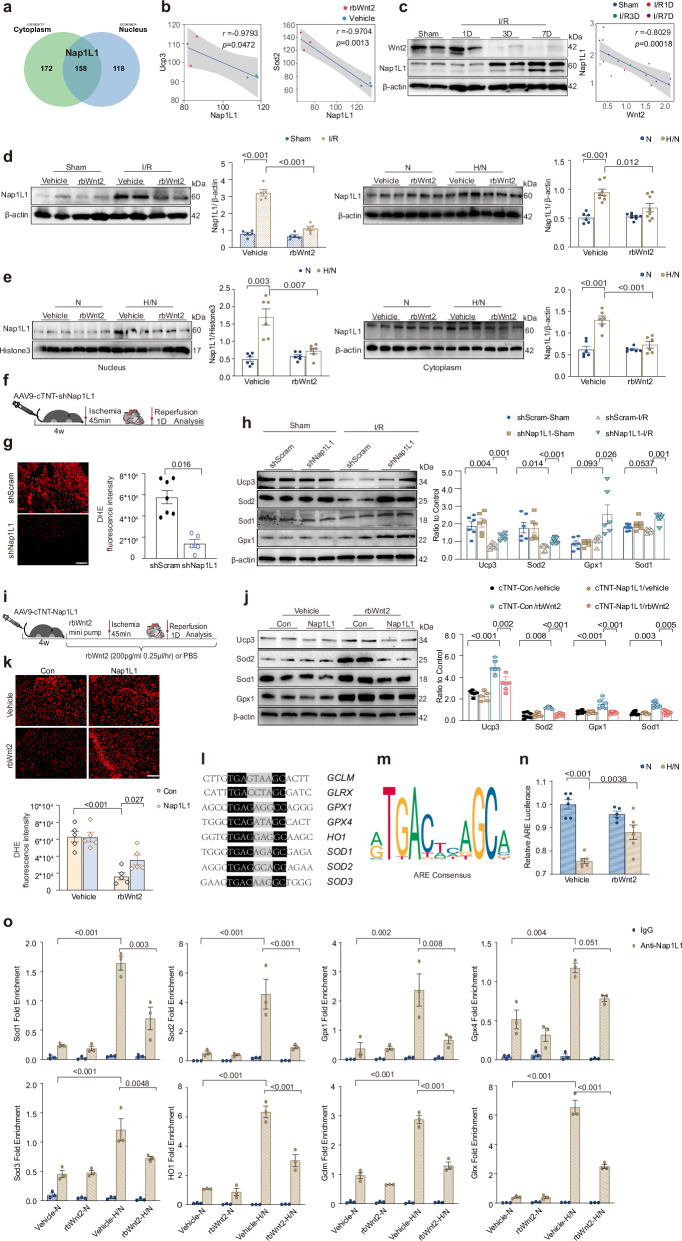
Wnt2 downregulates Nap1L1 to reactivate antioxidant gene transcription in I/R hearts. **a** Venn diagram and GO analysis of differentially expressed proteins in the cytoplasm and nucleus. **b** Bioinformatics analysis of proteomic profiling data: Scatter plots showing the correlation between antioxidant enzymes and Nap1L1 in the vehicle or rbWnt2 groups. Spearman’s r, exact two-sided P values, and 95% confidence intervals are shown. *n* = 3/group. **c** Western blot analysis of cardiac Wnt2 and Nap1L1 levels in mice subjected to I/R at different time points. Scatter plots showing the correlation between Wnt2 and Nap1L1 expression in sham or I/R hearts at the indicated time points. Spearman’s r, exact two-sided *P* values, and 95% confidence intervals are shown. *n* = 4/group. **d** Left panel: Western blot of Nap1L1 expression in I/R or sham mice treated with rbWnt2 or PBS. *n* = 5/group. Right panel: Western blot analysis of Nap1L1 expression in adult mouse cardiomyocytes (AMCMs) cultured under H/N with rbWnt2 or PBS treatment. *n* = 6–8/group. **e** Nap1L1 expression in the nucleus (left panel) and cytoplasm (right panel) of AMCMs cultured under H/N with rbWnt2 or PBS treatment. *n* = 6/group. **f** Schematic of the experimental workflow: AAV9-cTnT-shNap1L1 was injected via the tail vein to knockdown Nap1L1 specifically in cardiomyocytes, followed by I/R. **g** Representative DHE-stained images of I/R-injured hearts from mice injected with AAV9-cTnT-shNap1L1 or AAV9-cTnT-Con via the tail vein. Scale bar: 100 μm. *n* = 5–6/group. **h** Western blot analysis of Ucp3, Sod2, Sod1, and Gpx1 levels in I/R-injured heart tissues from mice injected with AAV9-cTnT-shNap1L1 or AAV9-cTnT-Con via the tail vein. *n* = 6–7/group. **i** Schematic experimental workflow: AAV9-cTnT-Nap1L1 was injected via the tail vein to overexpress Nap1L1 specifically in cardiomyocytes pretreated with recombinant human Wnt2 protein (rbWnt2, 200 pg/mL, 0.25 μL/h) or PBS via chronic minipump infusion, followed by I/R. **j** Western blot analysis of Ucp3, Sod2, Sod1, and Gpx1 levels in I/R-injured heart tissues from mice injected with AAV9-cTnT-Nap1L1 or AAV9-cTnT-Con via the tail vein. *n* = 5–6/group. **k** Representative DHE-stained images of I/R-injured hearts from mice injected with AAV9-cTnT-Nap1L1 or AAV9-cTnT-Con via the tail vein. Scale bar: 100 μm. *n* = 5/group. **l**, **m** Consensus DNA-binding motifs of antioxidant response elements (AREs). **n** Luciferase reporter assay in HEK293A cells transduced with a luciferase reporter driven by ARE promoters. *n*=5-6/group. **o** ChIP‒qPCR assay in HEK293A cells cultured under hypoxia/normoxia (H/N) or normoxia (N) and treated with rbWnt2 or PBS (vehicle), showing Nap1L1 or IgG occupancy at ROS-scavenging gene promoter fragments *n*. *n* = 3/group. All the data are expressed as the means ± SEMs

To elucidate the molecular mechanisms by which Wnt2 regulates ROS levels during I/R injury, we first assessed the activation of canonical Wnt/β-catenin signaling. Compared with control cardiomyocytes, H/N-treated cardiomyocytes presented reduced active β-catenin levels, but the rbWnt2 and vehicle groups presented no significant differences under H/N conditions (Supplementary Fig. 4a). These findings suggest that Wnt2-mediated cardioprotection operates independently of canonical Wnt/β-catenin signaling. Proteomic profiling revealed that differentially expressed proteins (DEPs) in rbWnt2-treated hearts were predominantly localized to the cytoplasm or nucleus, with 158 proteins overlapping both compartments (Fig. 5a). Further bioinformatics analysis of the proteomic profiling data revealed a trend toward a negative correlation between antioxidant enzyme levels (Ucp3, Sod2)and Nap1L1 (nucleosome assembly protein-like 1) expression (Fig. 5b). Notably, Nap1L1, a regulator of cardiomyocyte differentiation identified in our prior work,^18^ was downregulated 2-fold in the rbWnt2 treatment group and emerged as a key candidate. Nap1L1 was negatively correlated with cardiac Wnt2 levels during I/R progression (Fig. 5c). RbWnt2 treatment significantly attenuated the upregulation of Nap1L1 induced by I/R injury in mice or in AMCMs under H/N (Fig. 5d). Subcellular fractionation further demonstrated that H/N-induced Nap1L1 accumulation in both the nuclear and the cytoplasmic compartments, and these effects were significantly suppressed by rbWnt2 (Fig. 5e).

To establish whether Wnt2 mitigates I/R-induced ROS via Nap1L1 downregulation, we employed loss- and gain-of-function approaches in vitro and in vivo. Cardiomyocyte-specific Nap1L1 knockdown was achieved via adenoviral shRNA in AMCMs and AAV9-cTnT-shNap1L1 delivery in mice (Fig. 5f and Supplementary Fig. 4b). The downregulation of Nap1L1 phenocopied the antioxidant actions of rbWnt2, effectively normalizing cytosolic and mitochondrial ROS overproduction and increasing Ucp3, Sod1, Sod2 and Gpx1 expression in both H/N-induced AMCMs and post-I/R myocardial tissue (Fig. 5g, h and Supplementary Fig. 5a–c). Nap1L1 overexpression was mediated by AAV9-cTnT-Nap1L1 in vivo or adenoviral Nap1L1 transfection in vitro (Fig. 5i and Supplementary Fig. 4c). Under basal conditions, overexpression of Nap1L1 did not affect the expression of the aforementioned antioxidant enzymes (with the exception of Sod1) or lipid peroxidation markers (Acsl4) (Supplementary Fig. 5d). However, under I/R conditions, Nap1L1 overexpression blocked the ability of rbWnt2 to suppress ROS levels and abolished rbWnt2-mediated antioxidant protein induction in vivo and in vitro (Fig. 5j, k and Supplementary Fig. 5e–g). Further analysis revealed that the 9 bp consensus motif is a specific ARE (antioxidant response element) motif, which is a cis-regulatory element found in these promoter sites of ROS-scavenging genes (Fig. 5l, m). The activation or regression of the transcription of these antioxidant genes has been reported to be regulated by the interaction of transcription factors with AREs.^31^ We observed that rbWnt2 treatment attenuated the nuclear accumulation of Nap1L1 but improved the decrease in ARE-mediated luciferase activity after I/R (Fig. 5n). Chip-PCR revealed that H/N enhanced Nap1L1 occupancy at antioxidant gene promoters containing conserved AREs, and this binding was attenuated by Wnt2 treatment (Fig. 5o).

Our data suggested that Wnt2 reactivates the transcription of antioxidant genes to reduce ROS levels via the downregulation of Nap1L1 after I/R injury.

### Nap1L1 downregulation mediates the dual inhibition of apoptosis and ferroptosis by Wnt2

**Fig. 6:**
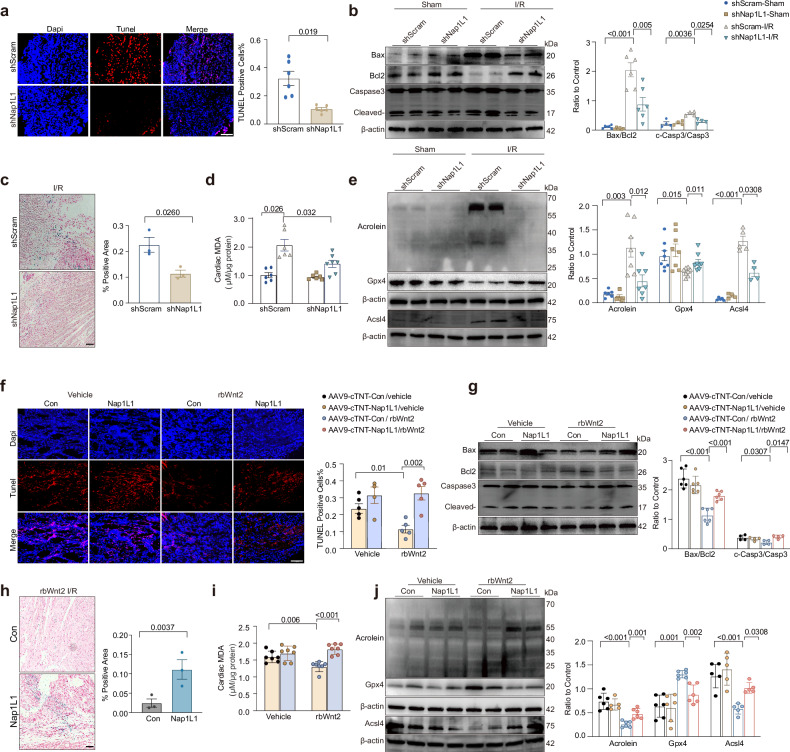
Wnt2 downregulation of Nap1L1 suppresses apoptosis and ferroptosis in I/R hearts. **a** Representative TUNEL staining of I/R hearts from mice injected with AAV9-cTnT-shNap1L1 or AAV9-cTnT-scramble via the tail vein. Scale bar: 100 μm. *n* = 6/group. **b** Western blot analysis of Bax, Bcl2, cleaved caspase-3, and caspase-3 levels in the I/R hearts of mice injected with AAV9-cTNT-ZsGreen-shNap1L1 or AAV9-cTNT-ZsGreen-NC via the tail vein. *n* = 4–6/group. **c** Representative images of Prussian blue staining in the I/R injury area. Scale bar: 100 μm. n = 3/group. **d** Malondialdehyde (MDA) levels in I/R hearts from the groups described in (**b**). *n* = 6–7/group. **e** Western blot of Acrolein, Gpx4 and Acsl4 in I/R hearts with or without Nap1L1 knockdown. *n* = 5–8/group. **f** TUNEL staining analysis of I/R-injured hearts from mice injected with AAV9-cTnT-Nap1L1 or AAV9-cTnT-control (AAV9-cTnT-Con) via the tail vein and treated with rbWnt2 or vehicle. Scale bar: 100 μm. *n* = 4–5/group. **g** Western blot analysis of the levels of the apoptosis-related proteins Bax, Bcl2, cleaved caspase-3, and caspase- 3 in I/R hearts from mice pretreated with AAV9-cTNT-Nap1L1 or AAV9-cTNT-control and those pretreated with rbWnt2 or vehicle. *n* = 4–6/group. **h** Representative images of Prussian blue staining in the I/R injury area. Scale bar: 100 μm. *n* = 3/group. **i** Malondialdehyde (MDA) levels in I/R hearts from the groups described in (**g**). *n* = 7/group. **j** Western blot of Acrolein, Gpx4 and Acsl4 expression in I/R tissues with or without Nap1L1 overexpression. *n* = 5–6/group. All the data are expressed as the means ± SEMs

To determine whether Wnt2 mediates the inhibition of apoptosis and ferroptosis by Nap1L1 post-I/R. Nap1L1 downregulation substantially attenuated the increase in the number of TUNEL-positive nuclei and the activation of caspase-3 in H/N-treated AMCMs and I/R-injured hearts, achieving efficacy comparable to that of rbWnt2 treatment (Fig. 6a, b and Supplementary Fig. 6a, b). Furthermore, the knockdown of Nap1L1 increased the viability of RSL3-treated AMCMs, decreased the levels of lipid peroxidation markers (Acsl4, acrolein and MDA) and iron deposition, and restored Gpx4 expression in I/R hearts or AMCMs under H/N (Fig. 6c–e and Supplementary Fig. 6c–e). Nap1L1 overexpression abolished the inhibitory effect of rbWnt2 on apoptosis during I/R injury in vivo and in vitro (Fig. 6f, g and Supplementary Fig. 6f, g). RbWnt2 increased the viability of AMCMs treated with the ferroptosis inducer RSL3, and this effect was attenuated by Nap1L1 overexpression (Supplementary Fig. 6h). Nap1L1 overexpression reversed the rbWnt2-mediated reduction in MDA/Acrolein/Acsl4 levels and iron accumulation and restored Gpx4 levels in both I/R hearts and H/N-stimulated cardiomyocytes (Fig. 6h–j and Supplementary Fig. 6i, j).

Collectively, these data establish Nap1L1 downregulation as a critical mechanism through which rbWnt2 mitigates oxidative damage and coordinates myocardial protection against apoptosis and ferroptosis during cardiac I/R injury.

### Regulation of Nap1L1 is critical for the cardioprotective effect of Wnt2 following I/R injury

**Fig. 7:**
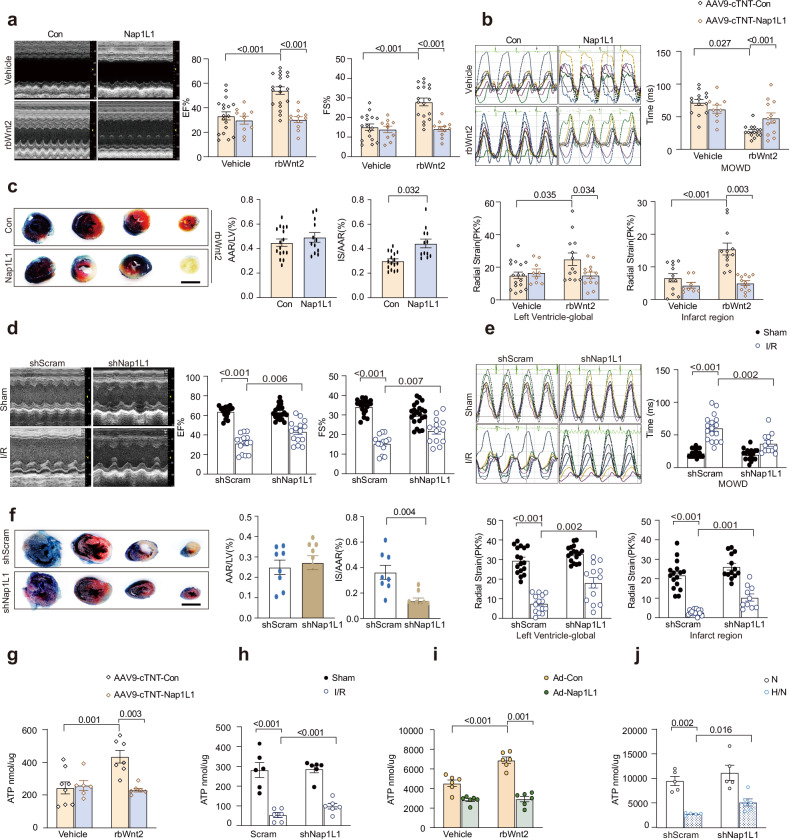
The regulation of Nap1L1 is critical for the cardioprotective effects of Wnt2 following I/R injury. **a** Representative M-mode echocardiograms and quantitative analysis of the left ventricular ejection fraction (EF) and fractional shortening (FS) in I/R model mice injected with AAV9-cTnT-Nap1L1 or AAV9-cTnT-control via the tail vein and infused with vehicle or rbWnt2. *n* = 12–17/group. **b** Radial strain analysis of the mice in the groups described in (**a**). Left panel: Representative radial strain curves. The colored lines represent the six standard myocardial regions. Right panel: Quantitative analysis of global radial strain, infarct area radial strain, and MOWD (maximum opposite wall delay). *n* = 8–17/group. **c** Evans blue and triphenyltetrazolium chloride (TTC) staining analysis of the infarct area in I/R model mice injected with AAV9-cTnT-Nap1L1 or AAV9-cTnT-control and infused with rbWnt2. *n* = 12–16/group. IS/AAR: Infarct size/area at risk (% of AAR). Scale bar: 3 mm. **d** Representative M-mode echocardiograms and quantitative analysis of EF and FS in I/R model mice preinjected with AAV9-cTnT-shNap1L1 (sh-Nap1L1) or AAV9-cTnT-shScramble (scramble) via the tail vein. *n* = 13–19/group. **e** Radial strain analysis of the mice in the groups described in (**d**). Left panel: Representative radial strain curves. Right panel: Quantitative analysis of global radial strain, infarct area radial strain, and MOWD. *n* = 10–18/group. **f** Evans blue and TTC staining analysis of the infarct area in I/R model mice injected with AAV9-cTnT-shNap1L1 (sh-Nap1L1) or AAV9-cTnT-shScramble (scramble). Scale bar: 3 mm. *n* = 8–9/group. IS/AAR: Infarct size/area at risk (% of AAR). **g** ATP levels in I/R hearts with or without Nap1L1 overexpression after rbWnt2 or PBS (vehicle) treatment. *n* = 6–8/group. **h** ATP levels in the infarct area of tissue with or without Nap1L1 knockdown. *n* = 6/group. **i** ATP levels in cultured adult mouse cardiomyocytes (AMCMs) after H/N with or without Nap1L1 overexpression under rbWnt2 or PBS (vehicle) treatment. *n* = 6/group. **j** ATP levels in AMCMs after H/N with or without Nap1L1 knockdown. *n* = 5/group. All the data are expressed as the means ± SEMs

To define the mechanistic role of Nap1L1 suppression in Wnt2-mediated cardioprotection against I/R injury, loss- and gain-of-function approaches in vitro and in vivo were used as previously described. Nap1L1 overexpression had few effects on heart function, including LVEF, LVFS, and radical and longitudinal strain (Supplementary Fig. 7a). Unlike in the control group, in cardiac Nap1L1-overexpressing mice, rbWnt2 failed to improve the LVEF and LVFS (Fig. 7a). Strain and strain rate analyses revealed that Nap1L1 overexpression significantly decreased PK% in global and infarcted areas in mice treated with rbWnt2. The maximum opposite wall delay (MOWD) significantly decreased over time in the rbWnt2-treated mice but increased in the Nap1L1-overexpressing mice (Fig. 7b). The longitudinal strain analysis results were consistent with the radial strain analysis results (Supplementary Fig. 7b). Notably, the rbWnt2-induced reduction in infarct size (TTC staining) was reversed by Nap1L1 overexpression (Fig. 7c). Next, we confirmed the beneficial effect of Nap1L1 downregulation during heart I/R injury. Nap1L1 silencing phenocopied rbWnt2 effects. Compared with control mice, shNap1L1-treated mice presented significantly greater LVEF, LVFS, and strain parameters post-I/R (Fig. 7d, e and Supplementary Fig. 7c). In addition, TTC staining revealed that, compared with control treatment, Nap1L1 knockdown markedly decreased the infarct size following I/R (Fig. 7f). ATP production was also examined in vivo and in vitro. Nap1L1 knockdown improved the reduced ATP level in AMCMs in H/N or I/R hearts, whereas Nap1L1 overexpression attenuated the increased ATP level induced by rbWnt2 in H/N AMCMs or I/R hearts (Fig. 7g–j).

Taken together, these findings demonstrated that the downregulation of Nap1L1 is a key player in the cardioprotective effect of rbWnt2 intervention in response to I/R injury.

### RbWnt2 acts on Lrp6 to promote Nap1L1 degradation by Trim11 in response to I/R

**Fig. 8:**
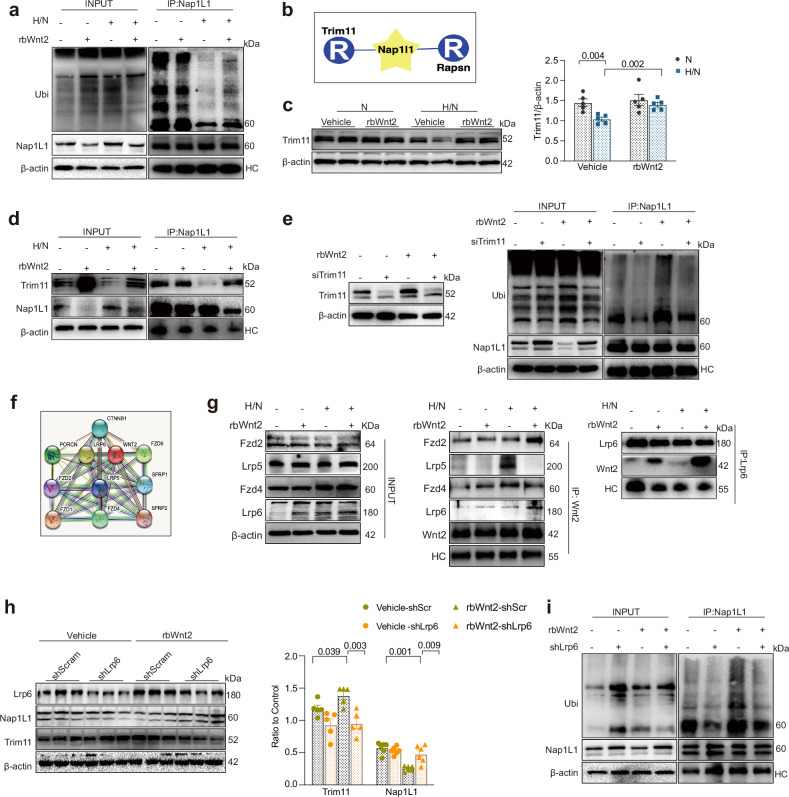
rbWnt2 acts on Lrp6 to promote Nap1L1 degradation via Trim11-mediated ubiquitination in response to I/R. **a** Analysis of Nap1L1 ubiquitination. Lysates from cultured cardiomyocytes treated with rbWnt2 or PBS (vehicle) under hypoxia/normoxia (H/N) conditions were immunoprecipitated with an anti-Nap1L1 antibody and immunoblotted with a ubiquitin antibody. **b** UbiBrowser (http://ubibrowser.ncpsb.org) was used to explore putative E3 ligases that could ubiquitinate Nap1L1. In the network view, a central node represents Nap1L1 (putative substrate), surrounded by nodes indicating predicted E3 ligases. **c** Western blot analysis of Trim11 expression in AMCMs from (**a**). *n* = 5/group. **d** Coimmunoprecipitation (co-IP) analysis of the Trim11-Nap1L1 interaction in cardiomyocytes from (**a**). Lysates were immunoprecipitated with an anti-Nap1L1 antibody and immunoblotted with an anti-Trim11 antibody. **e** Nap1L1 ubiquitination was analyzed by immunoprecipitation in cardiomyocytes transfected with si-Trim11 or si-scramble and treated with PBS or rbWnt2. Lysates were immunoprecipitated with an anti-Nap1L1 antibody and immunoblotted with a ubiquitin antibody. **f** The STRING database (https://cn.string-db.org) was used to analyze putative or confirmed proteins that interact with Wnt2. Nodes represent proteins, edges represent protein‒protein associations, and colored lines indicate interaction types. **g** Left and middle panel: the cell lysates treated with rbWnt2 or PBS as control cultured under H/N conditions were immunoprecipitated with anti-Wnt2 antibodies, and then immunoblotted with Fzd2, Fzd4, Lrp5 and Lrp6 antibodies. Right panel: lysates were immunoprecipitated with an anti-Lrp6 antibody and immunoblotted with an anti-Wnt2 antibody. **h** Western blot analysis of Nap1L1 and Trim11 expression in cardiomyocytes transfected with lenti-shScramble (shScramble) or lenti-shLrp6 (shLrp6) and treated with PBS or rbWnt2 under H/N. *n* = 4–6/group. **i** IP analysis of Nap1L1 ubiquitination in cardiomyocytes from (**h**). Lysates were immunoprecipitated with an anti-Nap1L1 antibody and immunoblotted with a ubiquitin antibody. All the data are expressed as the means ± SEMs

To investigate the underlying molecular mechanism responsible for the rbWnt2-mediated reduction in Nap1L1 expression in cardiomyocytes post-I/R, we evaluated the Nap1L1 ubiquitination level in cardiomyocytes treated with rbWnt2 or with PBS as a control to investigate whether the ubiquitination of Nap1L1 influenced rbWnt2 intervention. The experimental results demonstrated that the decrease in Nap1L1 ubiquitination after I/R was reversed by rbWnt2 treatment (Fig. 8a). Bioinformatics interrogation through UbiBrowser (http://ubibrowser.ncpsb.org) identified Trim11 and Rapsn as the top E3 ligase candidates for Nap1L1^32^ with high confidence (Fig. 8b). Subsequent validation revealed that rbWnt2 specifically reversed H/N-induced Trim11 downregulation (Fig. 8c) without modulating Rapsn expression dynamics (Supplementary Fig. 8a). Coimmunoprecipitation (co-IP) analysis revealed that the interaction between Trim11 and Nap1L1 was decreased in cardiomyocytes after H/N injury but was restored by rbWnt2 treatment (Fig. 8d). Furthermore, Trim11 silencing blocked the increased Nap1L1 ubiquitination mediated by rbWnt2 intervention in H/N cardiomyocytes (Fig. 8e). Together, these results provide explicit evidence that rbWnt2 supplementation promoted the degradation of Nap1L1 by Trim11 for cardiac protection following I/R.

Wnt2 is the ligand for members of the frizzled family or Lrp6/5 and functions in the canonical Wnt signaling pathway by activating transcription factors of the TCF/LEF family.^33^ To gain insight into which receptors mediate the cardiac protection of rbWnt2 against I/R injury, we analyzed the STRING database (https://cn.string-db.org) and found that Wnt2 could interact with multiple proteins (putative or confirmed), including Lrp5/6 and Fzd1/2/4/9 (Fig. 8f). Co-IP confirmed that the binding of Wnt2 and Lrp6 was increased by Wnt2 treatment in the control or H/N-AMCMs. In AMCMs under H/N, rbWnt2 strongly promoted the interaction of Wnt2 with Lrp6 or Fzd2 (Fig. 8g). Lrp6, a Wnt coreceptor, was shown to protect the heart against oxidative stress in our recent study.^34^ To confirm the involvement of Lrp6 in the ability of rbWnt2 to promote the ubiquitination of Nap1L1, we transfected cardiomyocytes with shRNA-Lrp6-expressing lentivirus (shLrp6) or shRNA-scramble lentivirus (control). Lrp6 silencing suppressed the increase in Trim11 expression and decrease in Nap1L1 expression induced by rbWnt2 in H/N cardiomyocytes (Fig. 8h). However, silencing Fzd2 did not affect the Wnt2 mediated-downregulation of Nap1L1 in H/N AMCMs (Supplementary Fig. 8b). Consistent with these findings, the rbWnt2-induced increase in Nap1L1 ubiquitination was blocked by Lrp6 silencing in H/N cardiomyocytes (Fig. 8i).

**Fig. 9:**
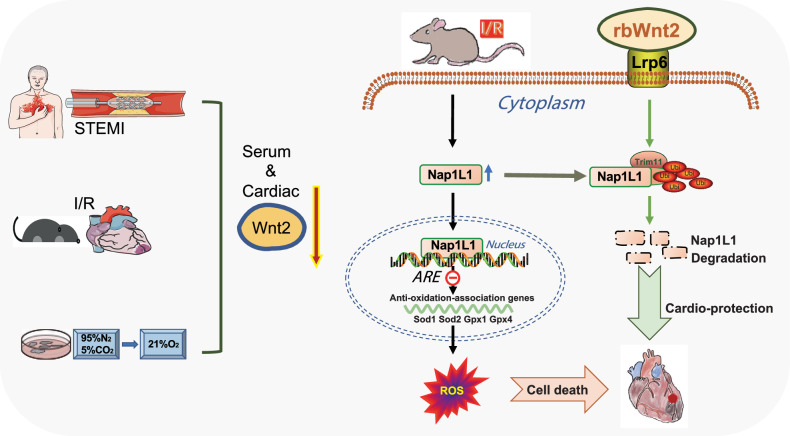
Wnt2 signaling attenuates myocardial I/R injury by orchestrating Lrp6/Trim11/Nap1L1-mediated redox homeostasis. Ischemia/reperfusion (I/R)-induced Wnt2 downregulation exacerbates myocardial injury through redox dysregulation, concurrently activating apoptosis and ferroptosis. Pharmacological Wnt2 restoration mitigates oxidative damage via the Lrp6/Trim11/Nap1L1 axis, driving Nap1L1 ubiquitination for proteasomal degradation and reactivating antioxidant gene transcription to suppress ROS. This tripartite mechanism establishes recombinant Wnt2 as a therapeutic agent that targets spatial oxidative stress dynamics in ischemic heart disease. This image has been created with Procreate Pocket, Microsoft Powerpoint and Adobe Illustrator. The image materials were obtained for free from Servier Medical ART and purchased from Taobao

Together, these orthogonal approaches conclusively demonstrate that Wnt2-Lrp6 signaling recruits Trim11 to orchestrate Nap1L1 ubiquitination degradation, constituting a druggable axis for cardioprotection against I/R injury (Fig. 9).

## Discussion

In this study, we demonstrated a progressive decline in serum and cardiac Wnt2 levels following ischemia/reperfusion (I/R) injury in both clinical cohorts and murine models. Recombinant Wnt2 (rbWnt2) administration significantly ameliorated I/R-induced damage, as evidenced by improved cardiac function, reduced infarct size, preserved ATP levels, and suppressed oxidative stress through the inhibition of mitochondrial and cytosolic reactive oxygen species (ROS) overproduction.

Wnt proteins, including Wnt2, are secreted by almost all metazoan cells; thus, serum Wnt2 is secreted from multiple tissues, and the data revealed that I/R decreased wnt2 levels in heart tissue but not in other tissues, such as the liver, lung or kidney (data not shown). These data suggested that decreased cardiac Wnt2 expression and secretion might lead to low levels of Wnt2 in the serum following I/R. Further analysis demonstrated that decreased Wnt2 expression and secretion in cardiomyocytes and cardiac fibroblasts might be one of the reasons for the reduction in serum Wnt2 after reperfusion. The detailed molecular mechanisms need further study. Mechanistically, rbWnt2-mediated cardioprotection operates independently of canonical Wnt/β-catenin signaling. Nap1L1 was identified as a key downstream effector. I/R-induced upregulation of Nap1L1, which otherwise binds to antioxidant response elements (AREs) within the promoters of ROS-scavenging genes (e.g., SODs, GPXs, and UCP3), thereby repressing their transcription. This repression was reversed by rbWnt2 via Trim11-mediated ubiquitination and subsequent degradation of Nap1L1, a process facilitated by the interaction between Wnt2 and the Lrp6 receptor. Collectively, these findings identify the Wnt2–Lrp6/Trim11–Nap1L1 axis as a promising therapeutic target for attenuating I/R injury by increasing antioxidant gene expression and concurrently decreasing cardiomyocyte death.

Wnt2, a secreted glycoprotein and a member of the Wnt family, is an important component of the Wnt signaling pathway. Wnt2 usually activates the classical Wnt/β-catenin signaling pathway, exerting its effects through autocrine or paracrine mechanisms, and is closely related to biological processes such as embryonic development and the occurrence and development of tumors.^35,36^ In Parkinson’s disease (PD) transgenic Drosophila, Wnt2 overexpression inhibited oxidative stress, as evidenced by the downregulation of ROS and MDA production and the increase in manganese superoxide dismutase (MnSOD), which is independent of β-catenin signaling.^15^ Our previous study revealed increased serum levels of Wnt2 and Wnt4 in AMI patients compared with healthy controls.^14^ The current data revealed that the expression of only Wnt2 but not Wnt4 was decreased in AMI patients post-PCI and that lower Wnt2 levels were correlated with worse I/R injury. This finding implies that Wnt2 may play a protective role and that its reduction could exacerbate injury by diminishing protective mechanisms. In murine I/R models, Wnt2 supplementation exhibited robust cardioprotective effects by greatly reducing the production of reactive oxygen species (ROS), malondialdehyde (MDA) and lipid peroxides following I/R injury. However, the molecular mechanisms underlying the effects of Wnt2 have not yet been elucidated.

Reperfusion-induced ROS production causes oxidative stress, which is responsible for most I/R injuries. Severe or sustained ROS-mediated damage can lead to programmed forms of cell death, including ferroptosis, apoptosis, pyroptosis, oxeiptosis, and necrosis. Cardiomyocyte death is referred to as lethal reperfusion injury, the irreversibility of which is central to permanent cardiac damage. This process drives heart failure through two pathways: acute infarction expansion (accounting for 50% of ischemia–reperfusion injuries) and chronic maladaptive ventricular remodeling.^37^ Among them, we focused on apoptosis and ferroptosis. Apoptosis and ferroptosis have been implicated in the critical pathological process associated with I/R injury. Excessive ROS trigger the opening of the mitochondrial PTP, the release of mitochondrial cytochrome c, and the activation of a caspase cascade, eventually leading to apoptosis.^38^ I/R induces cardiomyocyte apoptosis via intrinsic and extrinsic pathways. Notably, rbWnt2 intervention selectively attenuated intrinsic apoptosis, whereas it had no appreciable effect on the extrinsic apoptosis pathway. In addition to caspase-dependent apoptosis, caspase-independent apoptosis is also involved in ischemic-reperfusion injury.^39^ Whether Wnt2-induced protection is mediated by a caspase-independent pathway requires further study. Moreover, excess ROS-induced lipid peroxidation plays a critical role in cardiomyocyte ferroptosis following I/R. Ferroptosis, an iron-dependent programmed cell death mechanism driven by lethal lipid peroxide accumulation,^22^ has recently emerged as a critical contributor to I/R injury and a promising therapeutic target.^40^ Cellular ROS levels are balanced by ROS production through aerobic metabolism and the scavenging or degradation of ROS to nontoxic molecules by antioxidant defense systems. Mitochondrial ROS (mROS) are the main source of cellular ROS.^41^ During reperfusion, elevated ROS production is counteracted by endogenous antioxidants—including enzymes (SOD, catalase, GPx) and nonenzymatic components (vitamins/analogs)—which protect the heart from oxidative damage.^42^ Sod, a metalloenzyme, catalyzes the dismutation of superoxide anions into hydrogen peroxide and oxygen. Gpxs, which are highly expressed in the heart, exert greater protective effects than Sod against oxidative damage since they scavenge excess peroxides and limit the formation of hydroxyl radicals.^42^ UCPs, located in the inner membrane of mitochondria, regulate adenosine triphosphate and ROS generation. Our data revealed that Wnt2 promoted Ucp3 expression post-I/R. Ucp3 is highly expressed in the heart.^43^ Ucp3 provides cardiac protection by preventing myocardial necrosis and contractile dysfunction and alleviating mitochondrial calcium overload and mitochondrial ROS production during IR injury.^41,44^ The present data revealed that Wnt2 promoted the mRNA and protein levels of Sod1, Sod2, Gpx1, Gpx4, and Ucp3, suggesting that Wnt2 activates the production of these antioxidant enzymes to relieve the oxidative stress caused by free radicals following I/R.

Proteomic and functional analyses revealed that rbWnt2 suppresses I/R-induced Nap1L1 upregulation independently of canonical Wnt/β-catenin signaling, which otherwise represses the transcription of antioxidant genes (e.g., SODs, GPXs, and UCP3) by binding to ARE motifs in their promoters. Nap1L1 belongs to the family of nucleosome assembly proteins, which contribute to cell DNA replication and chromatin regulation by participating in the assembly and disassembly of nucleosomes.^45^ Nap1L1 is widely reported to be increased in multiple tumors and stimulates tumor progression, and elevated Nap1L1 is considered an unfavorable prognostic biomarker for several various tumors.^46,47^ Our previous study revealed that the downregulation of Nap1L1 promotes cardiomyocyte differentiation via the regulation of notch signaling in murine iPSCs.^18^ Cheng Lv et al. reported that NAP1L1p. D349E variant in HCM patients, and the variant promotes cardiac hypertrophy by activating the cGAS-STING-IFN pathway.^48^ However, there are few reports about the effect of Nap1L1 in cardiomyocytes in response to I/R. Our present study suggested that I/R increased nuclear Nap1L1 and its ability to bind to specific promoter sites of ROS-scavenging genes to inhibit their transcription and thus cause cellular ROS accumulation, which could be largely reversed by rbWnt2 treatment. Our data revealed that Wnt2 inhibited apoptosis and ferroptosis to protect the heart from I/R injury via the downregulation of Nap1L1 and subsequently decreased ROS levels, and the knockdown of Nap1L1 improved cardiomyocyte apoptosis under hypoxia/normoxia (H/N). However, Cheng Lv et al. reported that knockdown of Nap1L1 induced apoptosis in neonatal rat cardiomyocytes (NRCMs), which is not consistent with our results. Cheng Lv et al. used neonatal cardiomyocytes transfected with siRNA-Nap1L1 to detect apoptosis under basic conditions, but we explored apoptosis in adult mouse cardiomyocytes (AMCMs) under hypoxia/normoxia (H/N), and these cells were transfected with adenoviral shRNA-Nap1L1. Thus, we speculate that the effects of Nap1L1 on cardiomyocyte apoptosis are dependent on the species or pathological conditions.

The therapeutic significance of Nap1L1 inhibition was unequivocally demonstrated through gain- and loss-of-function studies: Nap1L1 knockdown phenocopied the benefits of rbWnt2, whereas its overexpression abolished cardioprotection.

Wnt2 reduced the protein level of Nap1L1 but did not affect the mRNA level of Nap1L1 in hypoxic cardiomyocytes (data not show). These findings suggest that Wnt2 might promote the degradation of Nap1L1 in cardiomyocytes during I/R. UbiBrowser analysis indicated that two enzymes, Rapsn and Trim11, could interact with Nap1L1. Our data demonstrated that Wnt2 attenuated the decreased expression of Trim11 but did not affect the level of Rapsn in hypoxic cardiomyocytes. Trims are ubiquitin E3 ligases characterized by an N-terminal TRIM/RBCC motif that mediate the degradation of aberrant proteins or normal regulatory proteins.^49^ Trim11 promotes the clearance of misfolded proteins, which contributes to reduced oxidative stress and subsequent tumorigenesis.^50^ The present data revealed that Wnt2 acted on Lrp6 to increase the expression of Trim11 and promote the degradation of Nap1L1 following I/R. Wnt ligands usually act on Fzd receptors or the coreceptors Lrp5/6 to transduce signals and exert their biological actions. Our data showed that Wnt2 administration enhanced the interaction of Wnt2 with Lrp6 or Fzd2 following I/R injury. However, further analysis revealed that Wnt2 increased the expression of Trim11 and the ubiquitination of Nap1L1 through Lrp6 rather than Fzd2 in cardiomyocytes under H/N. These data suggest that Lrp6/Nap1L1/Trim11, which is beyond the canonical Wnt/β-catenin pathway, is specifically activated by Wnt2 for cardiac protection in the I/R model.

Wnt2 signaling exhibits stage-dependent duality in cardiac pathophysiology. Yin et al. reported that Wnt2 and Wnt4 promoted cardiac fibrosis by interacting with Lrp6 and Frz4 or Frz2 and subsequently activated β-catenin signaling during chronic MI. These findings suggested that Lrp6-Frz/β-catenin signaling-driven profibrotic gene expression is the common downstream pathway of Wnt2 and Wnt4 in AMI. In the present study, Wnt2 did not affect the inhibition of β-catenin induced by I/R. Therefore, we identified a Wnt2-Lrp6-Nap1L1-antioxidant cardioprotective axis independent of β-catenin signaling during early I/R injury. In addition, Wnt4 did not significantly change after I/R compared with that in the sham group, and Wnt4 treatment neither suppressed the expression of Nap1L1 nor restored the expression of antioxidant enzymes after I/R (data not shown). These data suggest that Wnt2 specifically activates the Lrp6/Trim11/Nap1L1 axis to protect cardiomyocytes from death during I/R injury. In addition to cardiomyocyte death, the Wnt pathway is engaged in other I/R-associated processes, including fibrosis, inflammatory responses, angiogenesis and cardiac hypertrophy. Further analysis revealed that supplementation with rbWnt2 did not further activate fibroblasts after I/R (data not shown), which revealed that the molecular mechanisms of fibrosis involving I/R injury might differ from those in the MI model, although both are ischemic injuries. Temporal Wnt2 dynamics revealed acute-phase depletion (1 week post-I/R) followed by recovery (2 weeks), which correlated with its early antiapoptotic function. We propose a biphasic model: Wnt2 prevents cardiomyocyte death acutely post-I/R but may exacerbate fibrosis in chronic MI via sustained elevation. Given its dual regulation of oxidative stress (Lrp6-Nap1L1) and fibrotic pathways, temporary rbWnt2 delivery during PCI is advocated to maximize acute protection while avoiding profibrotic effects. It has been reported that Wnt2 inhibits enteric bacterium-induced inflammation in intestinal epithelial cells.^51^ Restoring Wnt2-FZD9-β-catenin signaling reduces colitis severity in ulcerative colitis (UC) in murine models.^52^ During cerebral I/R, the activation of Wnt/β-catenin signaling induces the proliferation of vascular endothelial cells to promote angiogenesis.^10^ Whether Wnt2 protects the heart from I/R through the inhibition of inflammation or the activation of angiogenesis needs further study. This is a limitation of our study. In addition, we generated an I/R mouse model via ischemia for 45 min followed by reperfusion. Due to the short duration of ischemia, we delivered rbWnt2 into the mice before ischemia and observed the cardioprotection of Wnt2 following I/R. To be consistent with a clinically relevant scenario of I/R, we will study these factors in large animals, such as pigs, via PCI with the injection of Wnt2 into the coronary artery at the onset of reperfusion to explore the therapeutic potential of Wnt2.

In conclusion, this work redefines Wnt2 as a master regulator of I/R injury through Nap1L1-mediated transcriptional reprogramming, providing a mechanistic foundation for targeting redox dysregulation and multiple forms of cell death in post-I/R cardiomyopathy.

## Materials and methods

### Protein extraction and immunoblotting

Tissue protein and cell protein were extracted from the I/R injury area and cardiomyocytes, respectively, via RIPA buffer supplemented with a protease inhibitor cocktail (# P1005 Beyotime, China). The total protein concentration was determined via a BCA protein assay (#P0010S Beyotime, China). Equal amounts of extracted proteins were separated via 10% or 12.5% SDS‒PAGE according to their molecular weight, transferred to 0.2 μm PVDF membranes (R1CB66021 Millipore, Germany), and subsequently blocked with blocking buffer. All the membranes were incubated overnight at 4 °C with the following primary antibodies: anti-Nap1L1 Polyclonal Antibody (1:1000 14898-1-AP Proteintech, USA); anti-Wnt2/IRP Antibody (1:1000 ab109222 Abcam, UK); anti-Ucp3 (1:1000 PA1--24895 Thermo Fisher, USA) Bax (1:1000 #2772 Cell Signaling Technology, USA); anti-Bcl-2; polyclonal antibody (1:1000 26593-1-AP Proteintech, USA); anti-Caspase-3 antibody and cleaved caspase-3 rabbit mAb (1:1000 #9664 #9662 Cell Signaling Technology, USA); anti-SOD2/MnSOD (acetyl K68)[EPVANR2] (1:1000 ab137037 Abcam, UK); anti-superoxide Dismutase 1 [EP1727Y] (1:1000 ab51254 Abcam, UK); anti-Glutathione Peroxidase 1 (1:1000 ab22604 Abcam, UK); anti-Glutathione Peroxidase 4[EPNCIR144] (1:1000 ab125066 Abcam, UK); and anti-Acrolein antibody (1:1000 ab240918 Abcam, UK, WNT4 Polyclonal antibody (1:500 14371-1-AP Proteintech, USA); Acsl4 (1:1000 sc-271800 Santa Cruz Biotechnology, USA)；Histone H3 (1:1000 17168-1-AP Proteintech, USA); Lrp6 (1:1000 #3395 Cell Signaling Technology, USA); Collagen I (1:1000 67288-1-Ig Proteintech, USA); Caspase-8 (1:1000 A19549 Abclona, China); Cleaved Caspase-8 (1:1000 A27958 Abclona, China); Phospho-RIPK1 (Ser161) Monoclonal antibody (1:1000 66854-1-Ig Proteintech, USA); RIPK1-Specific Polyclonal antibody (1:1000 17519-1-AP Proteintech, USA); RIPK3 Polyclonal antibody (1:1000 17563-1-AP Proteintech, USA); Phospho-RIP3 (Thr231/Ser232) (E7S1R) Rabbit mAb (1:1000 #91702 Cell Signaling Technology, USA); Non-phospho (Active) β-Catenin (Ser33/37/Thr41) (D13A1) Rabbit mAb (1:1000 #8814 Cell Signaling Technology, USA); β-Catenin Antibody (1:1000 #9562 Cell Signaling Technology, USA); TRIM11 Polyclonal antibody (1:1000 10851-1-AP Proteintech, USA). The next day, all the membranes were incubated with HRP-conjugated secondary antibodies (1:5000) for 1 h at room temperature, followed by washing with TBST. The expression of proteins was clearly detected by a CCD imaging system (Bio-Rad, USA).

### Isolation of cardiac myocytes and fibroblasts from adult mouse hearts

The surfaces of the culture dishes were coated with laminin (1:100 #23017-015 Thermo Scientific, Singapore) in PBS overnight at 4 °C. Media and buffers were sterilized with a 0.2 μm filter and then handled under sterile conditions. Collagenase and protease XIV enzymes are required for isolation. The surgical area was cleaned with 75% ethanol following full anesthesia. The mouse chest was opened with skin forceps, and 7 ml of EDTA buffer was injected slowly and steadily into the base of the right ventricle within one minute with a 27 G hypodermic needle. Before the heart was removed, the ascending aorta should be clamped first, 10 ml of buffer without bubbles should be injected into the left ventricle near the apex with very little pressure, and the best flow rate should be maintained at 1 ml every 2 or 3 min. Next, 3 ml of perfusion buffer was injected into the left ventricle to clear the EDTA left in the heart chambers and the coronary circulation via the same perforation left by the previous injection. Again, the best pressure is to ensure that the heart is fully inflated with the slowest flow rate. After ensuring a clear heart chamber and coronary circulation without any blood, collagenase and protease XIV enzyme mixture buffer was injected into the ventricle through the original pore left by the previous injection. The digestion process is slow and sufficient to keep the heart fully inflated, and the flow rate can increase as the procedure progresses. Once satisfactory digestion was completed, the heart became soft and loose and could be separated into several very small pieces. To inhibit further enzymatic digestion, 5 ml of stop solution was added to the cell‒tissue suspension. The cell suspensions were passed through a 100 μm pore size strainer to remove undigested tissue debris, and the cardiomyocytes were allowed to settle by gravity for 20 min. The supernatant fraction contained nonmyocytes collected by centrifugation at 300 × *g* for 5 min, resuspended in preequilibrated fibroblast media and plated on culture surfaces. The fibroblasts were cultured in a 37 °C incubator. The media were changed after 24 h of culture and every 48 h thereafter. Cardiomyocyte sediments were resuspended sequentially in three calcium reintroduction buffers for 10 min at room temperature. The final myocyte pellet was resuspended in plating media and then plated on culture surfaces, after which the laminin solution was aspirated. After 1 h, the myocytes were incubated in culture media instead of plating media.

### Cell hypoxia/normoxia model

The cell culture media was transferred to hypoxia media and then incubated in a hypoxia incubator with an oxygen concentration lower than 0.1% for 1 h. After that, the cells were removed from the hypoxia incubator, and the hypoxia media was transferred to the culture media again and then cultured in a normal incubator.

### Cardiac ischemia/reperfusion model

Adult 8-week-old mice were anesthetized via inhalation of isoflurane. After being fully anesthetized, a small incision was made in the surgical area, which was previously cleaned with 75% ethanol, to visualize the third and fourth intercostal spaces. The chest was opened via mosquito forceps, and the heart was pushed out. A ligation was placed on the left anterior descending coronary artery 2 mm below the left auricular appendix via a 6–0 silk suture. The ST segment was elevated, as shown by an electrocardiogram, indicating that the ischemia model was constructed successfully. After 45 min of coronary occlusion, the ligation was released to allow reperfusion. The animals were analyzed at appropriate time points. During the surgical procedure and throughout the recovery period, a thermostatically controlled heating pad was used to maintain the body temperature of each mouse within its preoperative range. All animal experiments were approved by the Animal Ethics Committee of Zhongshan Hospital, Fudan University.

### Evans blue and triphenyltetrazolium chloride (TTC) staining

The left anterior descending coronary artery was occluded with a 6–0 silk suture again at the original location of ligation. Evans blue was injected into the left ventricle via the apex until the dye was visible in the cardiac veins. The heart was removed from the chest, frozen in liquid nitrogen very quickly, and then excised and sliced into 4–5 slices. All these slices were incubated in TTC at 37 °C for 10–20 min and transferred into formalin overnight. The next day, all the slices were scanned, and pictures were taken via a scanner. The area at risk and infarct sizes were quantified via ImageJ software (National Institutes of Health, Bethesda, MD, USA). The myocardial infarct size was investigated by calculating the percentage of the area at risk, and the area at risk was calculated as a percentage of the left ventricle.

### Terminal deoxynucleotidyl transferase dUTP nick end labeling (TUNEL) staining

Tissue and cardiomyocyte apoptosis were measured via a TUNEL assay (Beyotime #C1090, China). In accordance with the protocol, the samples were fixed with 4% paraformaldehyde for 30 min and then washed with PBS twice. Following treatment with 0.3% Triton for 5 min at room temperature, all the samples were incubated in TUNEL working medium for 1 h at 37 °C. After washing with PBS three times, fluorescence images were obtained via a confocal microscope (Olympus Corporation, Japan). Apoptotic cells were those with TUNEL-positive nuclei, and DAPI was used to label all the cell nuclei. The ratio of apoptotic cells was calculated as the percentage of total cells.

### Dihydroethidium (DHE) staining

Fresh frozen cardiac sections were incubated with 10 μM DHE solution for 30 min at 37 °C. After being washed with PBS, fluorescence images were taken via a confocal microscope (Olympus Corporation, Japan).

### Reactive oxygen species (ROS) assay

ROS at the cell level was detected with a Reactive Oxygen Species Assay Kit (Beyotime # S0033S, China). DCFH-DA, the active ingredient of the Reactive Oxygen Species Assay Kit, was diluted in culture media without BSA at a ratio of 1:1000 and used to cover the cells, which were incubated together for 30 min at 37 °C. After washing with PBS, fluorescence images were taken via a confocal microscope (Olympus Corporation, Japan).

### Mito-sox staining

The MitoSOX Red mitochondrial superoxide indicator for live-cell imaging was obtained from Invitrogen (USA M36008). The cells were incubated with 5 μM MitoSOX^TM^ reagent working solution for 10 min at 37 °C in the dark and then washed with PBS three times. Fluorescence images were taken with a confocal microscope (Olympus Corporation, Japan).

### siRNA transfection

siRNA was used to knock down Trim11 expression in cardiomyocytes in vitro. siRNA (10 nmol) and Lipofectamine® RNAiMAX Reagent (#13778075 Thermo Fisher, USA) were diluted with Opti-MEM and then mixed and incubated for 5 min at room temperature. Finally, the siRNA-lipid complex was added to the cells for 2 days.

### Osmotic minipump model

Seven- to eight-week-old C57BL/6 mice were randomly divided into two groups. Sterilized surgical instruments were prepared for surgery, and aseptic techniques were utilized throughout the entire procedure. Osmotic minipumps (Alzet, model 1002, USA) were filled with rbWnt2 in PBS (200 pg/ml), or PBS was used as a control. After the mice were anesthetized via inhalation of isoflurane, pumps were inserted subcutaneously on the back via a small incision that was cleaned with 75% ethanol. Next, the incisions were sutured and sterilized with 75% ethanol.

### Coimmunoprecipitation (Co-IP)

Total protein was extracted from cultured cardiomyocytes. A total of 1000 μg of total protein was incubated with rabbit IgG (#2729 CST, USA) or the indicated IP antibody at 4 °C overnight, and an appropriate amount of extracted protein was used as an input control. The next day, 40 μl of Protein A/G PLUS-Agarose was added to the IP mixture, which was subsequently incubated for 4 h at 4 °C. After centrifugation at 2500 rpm for 5 min at 4°C, the protein A/G PLUS-agarose beads were washed with PBS 3 times and then boiled with protein loading buffer for 5 min. Following centrifugation again, the supernatant was collected for western blot analysis.

### Transthoracic echocardiography analysis

Echocardiography was performed on mice subjected to I/R or a sham operation via a Vevo2100 ultrasound system (VisualSonics, Toronto, Canada). The mice were placed on an ECG platform in the supine position and anesthetized with 1–1.5% isoflurane, with their body temperature maintained at 37 °C and heart rate between 400–600 bpm. Then, B-mode images were obtained from the left parasternal long axis view, whereas M-mode images were captured at the midpapillary muscle level. All the measurements were double-blinded. The endocardial borders were traced via Vevo offline analysis software for at least three consecutive stable cardiac cycles on the basis of M-mode images. Then, the ejection fraction (EF), fractional shortening (FS), end-diastolic volume (EDV) and end-systolic volume (ESV) were calculated to assess left ventricle function. For M-mode images, radial and longitudinal strains were estimated to detect subtle global dysfunction via a speckle tracking algorithm. Regional dyssynchrony was measured by the maximum opposite wall delay during the motion of 6 standard anatomic segments in the left ventricle.

### ELISA

The concentration of Wnt2 was quantified via ELISA kits (#SEL820HuCloud Clone Corp China) according to the manufacturer’s protocols. Briefly, after the wells of a 96-well plate were washed, 100 μL of standard/sample was added, and the mixture was incubated for 2 h at 37 °C. The plate was then washed, and a biotin-conjugated Wnt2 antibody was added to each well and incubated for 1 h at 37 °C. The mixture was aspirated and washed with 350 μL of 1× wash solution in each well via a squirt bottle, multichannel pipette, manifold dispenser or autwasher, after which the mixture was incubated for 1–2 min. The remaining liquid from all the wells was removed completely by snapping the plate onto absorbent paper. In total, the samples were washed 3 times. After the last wash, any remaining wash buffer was removed by aspirating or decanting. The plate was inverted and blotted against absorbent paper. Streptavidin-HRP was added for 30 min at 37 °C. Finally, the reaction was stopped, and the optical density was measured accordingly.

### Real-time quantitative polymerase chain reaction

RNA was extracted from the area subjected to I/R injury using TRIzol reagent (B511311, Sangon Biotech China), and cDNA was synthesized with a PrimeScript RT-PCR Kit (R0036A Takara Bio Japan). qRT-PCR was carried out using 2 × AceQ qPCR SYBR Green Master Mix (Q121 Vazyme China) in accordance with the manufacturers’ protocols. Relative quantification of mRNA within the samples was performed via the 2−ΔΔCt method, and the results were normalized relative to the β-actin mRNA level.

### TMT6-based proteomics analysis of mice

The I/R areas of left ventricular tissues from rbWnt2- and vehicle-treated mice were collected to identify the abundances of proteins via the TMT6-based proteomics technique. In brief, total protein from the I/R areas was concentrated and extracted, and the concentration was measured with a BCA kit (Beyotime). After being subjected to enzymolysis, the lyophilized samples were labeled with TMT6 reagent. The labeled samples were combined and dried in vacuo for LC‒MS/MS analysis. To identify and quantify proteins, the abundance data were analyzed via R (v4.1.1). We considered proteins with a fold change (FC) > 1.5 or <0.67 and a *p* value < 0.05 to be differentially expressed between the two groups. The differentially expressed proteins were further processed for functional analysis via the Kyoto Encyclopedia of Genes and Genomes (KEGG) database^53^ via the clusterProfiler (v4.0)^54^ package.

### Study population

Patients (aged 40–85 years) with acute myocardial infarction subjected to percutaneous coronary intervention (PCI) were enrolledfrom Zhongshan Hospital of Fudan University from November 2019 to January 2020. The diagnoses were based on the “2018 ESC/ACC/AHA/WHF Guidelines for Fourth universal definition of myocardial infarction (2018)”.^55^ Patients with previous MI, severe heart failure, advanced liver or renal diseases, chronic or acute infections, autoimmune disease, poorly controlled diabetes or hypertension, myocarditis or cancer were excluded. The characteristics of the enrolled patients are shown in Table S1 in Supplementary Materials. The study protocol was approved by the Ethics Committee of Zhongshan Hospital, Fudan University (approval number: B2021-312R).

### Adeno-associated virus 9 production and animal injection

The shNap1L1-adeno-associated virus 9 (AAV9) with the cTnT promoter was generated and injected into 7-week-old mice via the tail vein to silence NaplL1 expression in cardiomyocytes. The siRNA sequence was subcloned and inserted into the GV478 vector to produce the shNaplLl plasmid by Hanheng Biotechnology (Shanghai, China). HEK-293Tcells were cultured in DMEM supplemented with 10% FBS. Briefly, cultured HEK-293Tcells were transfected with pds-AAV9-U6 plasmids containing shNap1L1 (5’CCGGCGATCCAGACTATGACCCAAACTCGAGTTTGGGTCATAGTCTGGATCGTTTTTG-3’), pHelper and pAAV-RC plasmids via the conventional CaCl2 transfection method. The cells and culture media were harvested and collected after 72 h of incubation. After centrifugation, the supernatant was collected and incubated with DNase (Benzonase, Merck) for 30 min. The AAV was purified by CsCl-gradient sedimentation by ultracentrifugation for 24 h in a CP100WX Centrifugal concentrator (Hitachi, Japan) at 175,000 × *g*. The purified AAVs were titrated via quantitative PCR and stored at −80 °C. Approximately 1.0 × 10^12^ virus particles were injected into the tail veins of male mice 3 weeks before sham or I/R surgery. Nap1L1-adeno-associated virus9 with the cTnT promoter was produced via a similar method.

### Adenovirus construction and transfection

To generate adenoviruses to silence Nap1L1 expression, a siRNA sequence (5’CCGGCGATCCAGACTATGACCCAAACTCGAGTTTGGGTCATAGTCTGGATCGTTTTTG-3’) was subcloned to produce the shNap1L1 plasmid by Hanheng Biotechnology (Shanghai, China). HEK-293A cells were cultured and transfected with the shNaplLl plasmid via the CaCl2 transfection method. The cells and culture media were harvested, thawed and frozen, followed by a 72-h incubation. These cells and medium were added to the HEK-293A cells again, which was repeated several times. The mild virus was purified via CsCl gradient sedimentation. The purified viruses were stored at −80 °C. Cardiomyocytes were exposed to adenovirus for 6 h, after which the culture medium was replaced. Adenoviruses overexpressing Nap1L1 were produced via a similar method.

### Lentiviral construction and transfection

Lentiviral vectors containing pLKO.1 shRNA-LRP6 and nontargeting shRNA control vectors were obtained from Thermo Fisher Scientific, Inc. (Waltham, USA). The pLKO.1 shRNA-LRP6, psPAX2 and pMD2. G (lentiviral packaging plasmids), were transfected into 293T cells to generate shRNA lentiviral particles via FuGENE 6. The shRNA-scramble pLKO.1 vector and packaging plasmids were transfected into 293T cells as controls. The Lrp6 sequence from Lrp6 PCS2 was cloned and inserted into the pCDH plasmid to generate Lrp6 lentiviral particles. The GFP-pCDH plasmid was used as a control. Lentivirus particles were harvested twice following 72 h of incubation. Cardiomyocytes were infected with culture medium containing lentiviral supernatant for 36 h, and Lrp6 expression was examined via western blot analysis.

### Chromatin immunoprecipitation

Nap1L1 and IgG antibodies were used for chromatin immunoprecipitation (ChIP). The cells were treated with 1% formaldehyde at 37 °C for 10 min, and 2.5 M glycine was then added to terminate the reaction. After the cells were rinsed three times with cold PBS, they were harvested in lysis buffer. Ten percent of the lysates were taken as 2% input, and the rest were incubated with appropriate amounts of Nap1L1 and IgG antibodies overnight at 4 °C, followed by the addition of 30 µl of protein A/G magnetic beads/salmon sperm DNA for 1 h at 4 °C. After the samples were washed five times with wash buffer, the lysates were heated at 65 °C for at least 4 h to reverse the covalent histone‒DNA bonds. The DNA was extracted, and real-time PCR was performed.

### Luciferase reporter gene assay

pHBLuc-ARE was synthesized by Hanheng Biotechnology (Shanghai, China) and transfected into cells. Firefly luciferase activity was detected via a luciferase reporter gene system purchased from Promega Corporation (Madison, USA). The luciferase activity was correlated with the protein concentration.

### Histochemical detection of iron via Prussian blue staining of paraffin-embedded tissue sections

The detection of ferric iron was performed on formalin-fixed, paraffin-embedded (FFPE) tissue sections (4–5 μm thick) via Perl’s Prussian blue stain. Briefly, the sections were deparaffinized in xylene and rehydrated through a graded ethanol series (100%, 95%, and 70%), followed by a rinse in deionized water. The slides were then incubated in freshly prepared Perl’s working solution—a 1:1 mixture of 2% potassium ferrocyanide and 2% hydrochloric acid—for 20–30 min at room temperature. After thorough washing in deionized water, the sections were counterstained with 0.5% neutral red for 1–2 min (or Nuclear Fast Red for 5 min) and rinsed again. Finally, the sections were dehydrated through a graded ethanol series, cleared in xylene, and mounted with synthetic resin. For quality control, positive control tissues (e.g., liver with known iron deposition) and negative controls (either omission of Perl’s solution or use of iron-free tissue) were included in each staining batch. Prussian blue-positive deposits were identified as bright blue granules, while the nuclei and background tissue appeared pink or red.

### Statistical analysis

All data error bars are presented as the means ± SEMs, and *P* values are presented as exact values. Student’s *t* test or the Mann‒Whitney U test was used to compare data between 2 groups, and 2-way ANOVA with the Bonferroni post hoc correction was used to compare >2 groups for normally distributed data. *P* < 0.05 was considered statistically significant. All the data were tested for normality and equal variance. Graphs were generated via GraphPad Prism v 8.0 software, and the statistical analysis was performed via SPSS.

## Supplementary information

Supplementary Materials for Nucleosome assembly protein-like 1 degradation-dependent novel cardioprotection mechanism of Wnt2 against ischemia‒reperfusion injury

## Data Availability

All the data generated or analyzed during this study are available from the corresponding authors.

## References

[1] Global burden of 87 risk factors in 204 countries and territories, 1990–2019: a systematic analysis for the Global Burden of Disease Study 2019. *Lancet*. **396**, 1223–1249 (2020). 10.1016/S0140-6736(20)30752-2 PMC7566194 33069327

[2] Yellon, D. & Hausenloy, D. Myocardial reperfusion injury. *N. Engl. J. Med.* **357**, 1121–1135 (2007). 17855673 10.1056/NEJMra071667

[3] Stefano, T. & Antonio, A. The NLRP3 inflammasome in acute myocardial infarction. *Nat. Rev. Cardiol.* **15**, 203–214 (2018). 29143812 10.1038/nrcardio.2017.161

[4] Sang-Bing, O. et al. The mitochondrial permeability transition pore and its role in myocardial ischemia reperfusion injury. *J. Mol. Cell Cardiol.* **78**, 23–349 (2014). 25446182 10.1016/j.yjmcc.2014.11.005

[5] Gustafsson, A. B. & Gottlieb, R. A. Heart mitochondria: gates of life and death. *Cardiovasc Res.* **77**, 334–343 (2007). 18006487 10.1093/cvr/cvm005

[6] Xuexian, F. et al. Ferroptosis as a target for protection against cardiomyopathy. *Proc. Natl. Acad. Sci. USA* **116**, 2672–2680 (2019). 30692261 10.1073/pnas.1821022116 PMC6377499

[7] Clevers, H. Wnt/beta-catenin signaling in development and disease. *Cell* **127**, 469–480 (2006). 17081971 10.1016/j.cell.2006.10.018

[8] Cingolani, O. Cardiac hypertrophy and the Wnt/Frizzled pathway. *Hypertension* **49**, 427–428 (2007). 17210831 10.1161/01.HYP.0000255947.79237.61

[9] Nusse, R. & Clevers, H. Wnt/β-catenin signaling, disease, and emerging therapeutic modalities. *Cell* **169**, 985–999 (2017). 28575679 10.1016/j.cell.2017.05.016

[10] Meng, Z. et al. Ischemia-reperfusion injury: molecular mechanisms and therapeutic targets. *Signal Transduct Target Ther.* **9**, 1–39 (2024). 38185705 10.1038/s41392-023-01688-x PMC10772178

[11] Guoming, Z. et al. LncRNA AZIN1-AS1 ameliorates myocardial ischemia-reperfusion injury by targeting miR-6838-5p/WNT3A axis to activate Wnt-β/catenin signaling pathway. *Vitr. Cell Dev. Biol. Anim.* **58**, 54–68 (2022). 10.1007/s11626-022-00646-1 35064471

[12] Kazuto, N. et al. Secreted frizzled-related protein 5 diminishes cardiac inflammation and protects the heart from ischemia/reperfusion injury. *J. Biol. Chem.* **291**, 2566–2575 (2015). 26631720 10.1074/jbc.M115.693937 PMC4742726

[13] Aisagbonhi, O. et al. Experimental myocardial infarction triggers canonical Wnt signaling and endothelial-to-mesenchymal transition. *Dis. Model Mech.* **4**, 469–483 (2011). 21324930 10.1242/dmm.006510 PMC3124051

[14] Chao, Y. et al. Elevated Wnt2 and Wnt4 activate NF-κB signaling to promote cardiac fibrosis by cooperation of Fzd4/2 and LRP6 following myocardial infarction. *EBioMedicine.* **74**, 107345 (2021). 10.1016/j.ebiom.2021.103745 PMC8669316 34911029

[15] Xia, S. et al. Wnt2 overexpression protects against PINK1 mutant‑induced mitochondrial dysfunction and oxidative stress. *Mol. Med. Rep.* **21**, 2633–2641 (2020). 32323790 10.3892/mmr.2020.11066

[16] Lee, J. et al. NAP1L1 accelerates activation and decreases pausing to enhance nucleosome remodeling by CSB. *Nucleic Acids Res.* **45**, 4696–4707 (2017). 28369616 10.1093/nar/gkx188 PMC5416873

[17] Li, L. et al. Knockdown of nucleosome assembly protein 1-like 1 promotes dimethyl sulfoxide-induced differentiation of P19CL6 cells into cardiomyocytes. *J. Cell Biochem.* **113**, 3788–3796 (2012). 22807403 10.1002/jcb.24254

[18] Gong, H. et al. Knockdown of nucleosome assembly protein 1-like 1 induces mesoderm formation and cardiomyogenesis via notch signaling in murine-induced pluripotent stem cells. *Stem Cells* **32**, 1759–1773 (2014). 24648372 10.1002/stem.1702

[19] Moussa, I. et al. Consideration of a new definition of clinically relevant myocardial infarction after coronary revascularization: an expert consensus document from the Society for Cardiovascular Angiography and Interventions (SCAI). *Catheter. Cardiovasc. Inter.* **62**, 1563–1570 (2013). 10.1016/j.jacc.2013.08.720 PMC3890321 24135581

[20] Bonora, M. et al. ATP synthesis and storage. *Purinergic Signal* **8**, 343–357 (2012). 22528680 10.1007/s11302-012-9305-8 PMC3360099

[21] Dixon, S. et al. Ferroptosis: an iron-dependent form of nonapoptotic cell death. *Cell* **149**, 1060–1072 (2012). 22632970 10.1016/j.cell.2012.03.042 PMC3367386

[22] Fang, X. et al. Ferroptosis as a target for protection against cardiomyopathy. *Proc. Natl. Acad. Sci. USA* **116**, 2672–2680 (2019). 30692261 10.1073/pnas.1821022116 PMC6377499

[23] Gao, M., Monian, P., Quadri, N., Ramasamy, R. & Jiang, X. Glutaminolysis and transferrin regulate ferroptosis. *Mol. Cell* **59**, 298–308 (2015). 26166707 10.1016/j.molcel.2015.06.011 PMC4506736

[24] Viswanathan, V. et al. Dependency of a therapy-resistant state of cancer cells on a lipid peroxidase pathway. *Nature* **547**, 453–457 (2017). 28678785 10.1038/nature23007 PMC5667900

[25] Hearse, D., Humphrey, S. & Chain, E. Abrupt reoxygenation of the anoxic potassium-arrested perfused rat heart: a study of myocardial enzyme release. *J. Mol. Cell Cardiol.* **5**, 395–407 (1973). 4355339 10.1016/0022-2828(73)90030-8

[26] Zweier, J., Flaherty, J. & Weisfeldt, M. Direct measurement of free radical generation following reperfusion of ischemic myocardium. *Proc. Natl. Acad. Sci. USA* **84**, 1404–1407 (1987). 3029779 10.1073/pnas.84.5.1404 PMC304438

[27] Murphy, M. How mitochondria produce reactive oxygen species. *Biochem. J.* **417**, 1–13 (2009). 19061483 10.1042/BJ20081386 PMC2605959

[28] Chen, Z. et al. Prevention of ischemia/reperfusion-induced cardiac apoptosis and injury by melatonin is independent of glutathione peroxdiase 1. *J. Pineal Res.* **46**, 235–241 (2009). 19141089 10.1111/j.1600-079X.2008.00654.x PMC2752734

[29] Hu, C. et al. Loss of thioredoxin 2 alters mitochondrial respiratory function and induces cardiomyocyte hypertrophy. *Exp. Cell Res.* **372**, 61–72 (2018). 30236513 10.1016/j.yexcr.2018.09.010

[30] Ardanaz, N. et al. Lack of glutathione peroxidase 1 accelerates cardiac-specific hypertrophy and dysfunction in angiotensin II hypertension. *Hypertension* **55**, 116–123 (2010). 19917877 10.1161/HYPERTENSIONAHA.109.135715 PMC3061336

[31] Lu, M., Ji, J., Jiang, Z. & You, Q. The Keap1-Nrf2-ARE pathway as a potential preventive and therapeutic target: an update. *Med. Res. Rev.* **36**, 924–963 (2016). 27192495 10.1002/med.21396

[32] Li, Y. et al. An integrated bioinformatics platform for investigating the human E3 ubiquitin ligase-substrate interaction network. *Nat. Commun.* **8**, 347 (2017). 28839186 10.1038/s41467-017-00299-9 PMC5570908

[33] Sousa, K. et al. Wnt2 regulates progenitor proliferation in the developing ventral midbrain. *J. Biol. Chem.* **285**, 7246–7253 (2010). 20018874 10.1074/jbc.M109.079822 PMC2844173

[34] Wang, Y. et al. Low density lipoprotein receptor related protein 6 (LRP6) protects heart against oxidative stress by the crosstalk of HSF1 and GSK3β. *Redox Biol.* **37**, 101699 (2020). 32905882 10.1016/j.redox.2020.101699 PMC7486456

[35] Staal, F. J., Luis, T. C. & Tiemessen, M. M. WNT signalling in the immune system: WNT is spreading its wings. *Nat. Rev. Immunol.* **8**, 581–893 (2008). 18617885 10.1038/nri2360

[36] Cody, J. et al. Wnt signaling in lung development, regeneration, and disease progression. *Commun. Biol.* **4**, 1–13 (2021). 34017045 10.1038/s42003-021-02118-w PMC8138018

[37] Yellon, D. M. & Hausenloy, D. J. Myocardial reperfusion injury. *N. Engl. J. Med.* **357**, 1121–1135 (2007). 17855673 10.1056/NEJMra071667

[38] Zhang, J. et al. ROS and ROS-Mediated Cellular Signaling. *Oxid. Med. Cell Longev.* **2016**, 4350965 (2016). 26998193 10.1155/2016/4350965 PMC4779832

[39] Kai, L. et al. Chlorogenic acid alleviates hepatic ischemia-reperfusion injury by inhibiting oxidative stress, inflammation, and mitochondria-mediated apoptosis in vivo and in vitro. *Inflammation.* **46**, 1061–1076 (2023). 36856879 10.1007/s10753-023-01792-8 PMC10188389

[40] Su, L. et al. Reactive oxygen species-induced lipid peroxidation in apoptosis, autophagy, and ferroptosis. *Oxid. Med. Cell Longev.* **2019**, 5080843 (2019). 31737171 10.1155/2019/5080843 PMC6815535

[41] Cadenas, S. Mitochondrial uncoupling, ROS generation and cardioprotection. *Biochim. Biophys. Acta Bioenerg.* **1859**, 940–950 (2018). 29859845 10.1016/j.bbabio.2018.05.019

[42] Tsutsui, H., Kinugawa, S. & Matsushima, S. Oxidative stress and heart failure. *Am. J. Physiol. Heart Circ. Physiol.* **301**, H2181–H2190 (2011). 21949114 10.1152/ajpheart.00554.2011

[43] Murray, A., Anderson, R., Watson, G., Radda, G. & Clarke, K. Uncoupling proteins in human heart. *Lancet* **364**, 1786–1788 (2004). 15541452 10.1016/S0140-6736(04)17402-3

[44] Ozcan, C., Palmeri, M., Horvath, T., Russell, K. & Russell, R. Role of uncoupling protein 3 in ischemia-reperfusion injury, arrhythmias, and preconditioning. *Am. J. Physiol. Heart Circ. Physiol.* **304**, H1192–H1200 (2013). 23457013 10.1152/ajpheart.00592.2012 PMC3652089

[45] Gill, J. et al. Crystal structure of malaria parasite nucleosome assembly protein: distinct modes of protein localization and histone recognition. *J. Biol. Chem.* **284**, 10076–10087 (2009). 19176479 10.1074/jbc.M808633200 PMC2665062

[46] Aydin, M., Gul, G., Kiziltan, R., Algul, S. & Kemik, O. Nucleosome assembly protein 1-like 1 (NAP1L1) in colon cancer patients: a potential biomarker with diagnostic and prognostic utility. *Eur. Rev. Med. Pharm. Sci.* **24**, 10512–10517 (2020). 10.26355/eurrev_202010_23403 33155206

[47] Le, Y. et al. NAP1L1 is a prognostic biomarker and contribute to doxorubicin chemotherapy resistance in human hepatocellular carcinoma. *Cancer Cell Int.* **19**, 228 (2019). 31516385 10.1186/s12935-019-0949-0 PMC6729091

[48] Lv, C. et al. Somatic NAP1L1 p.D349E promotes cardiac hypertrophy through cGAS-STING-IFN signaling. *Nat. Commun.* **16**, 3140 (2025). 40169585 10.1038/s41467-025-58453-7 PMC11961713

[49] Hatakeyama, S. TRIM proteins and cancer. *Nat. Rev. Cancer* **11**, 792–804 (2011). 21979307 10.1038/nrc3139

[50] Chen, L. et al. Enhanced degradation of misfolded proteins promotes tumorigenesis. *Cell Rep.* **18**, 3143–3154 (2017). 28355566 10.1016/j.celrep.2017.03.010 PMC5603913

[51] Xingyin, L. et al. Wnt2 inhibits enteric bacterial-induced inflammation in intestinal epithelial cells. *Inflamm. Bowel Dis.* **18**, 418–429 (2011). 21674728 10.1002/ibd.21788 PMC3294455

[52] Yingyan, L. et al. Shared pathogenic mechanisms of Parkinson's disease and ulcerative colitis: α7 nicotinic acetylcholine receptor as a potential therapeutic target. *Int. J. Biol. Macromol.* **319**, 145701 (2025). 40609942 10.1016/j.ijbiomac.2025.145701

[53] Kanehisa, M., Furumichi, M., Sato, Y., Ishiguro-Watanabe, M. & Tanabe, M. KEGG: integrating viruses and cellular organisms. *Nucleic Acids Res.* **49**, D545–D551 (2021). 33125081 10.1093/nar/gkaa970 PMC7779016

[54] Wu, T. et al. clusterProfiler 4.0: a universal enrichment tool for interpreting omics data. *Innovation* **2**, 100141 (2021). 34557778 10.1016/j.xinn.2021.100141 PMC8454663

[55] Thygesen, K. et al. Fourth universal definition of myocardial infarction. *Rev. Esp. Cardiol.* **72**, 72 (2019).30580786

