# Supplementary material for: Nucleosome assembly protein-like 1 degradation-dependent novel cardioprotection mechanism of Wnt2 against ischemia‒reperfusion injury
Source: Signal Transduct Target Ther. 2025 Dec 16;10:403. doi: 10.1038/s41392-025-02503-5 (PMC12705695; doi:10.1038/s41392-025-02503-5)
Supplement: Supplementary file 1 — Supplementary Materials for Nucleosome assembly protein-like 1 degradation-dependent novel cardioprotection mechanism of Wnt2 against ischemia‒reperfusion injury [file 41392_2025_2503_MOESM1_ESM.docx]

Supplementary Materials for

**Nucleosome assembly protein-like 1 degradation-dependent novel cardioprotection mechanism of Wnt2 against ischemia‒reperfusion injury**

Ying Wang1#, Liming Chen1#, Jinyi Lin 1#, Xi Liu1#, Kejia Jin1, Chenxing Huang1, Hao Wang1, Jianguo Jia1, Jian Wu1, Zhiwen Ding1, Pan Gao1*, Junbo Ge1, Hui Gong1*, Yunzeng Zou1, 2, 3*

Correspondence to: zou.yunzeng@zs-hospital.sh.cn

**This PDF file includes:**

Supplementary Tables S1 to S3

Supplementary Figures S1 to S8

**Table S1. The basic clinical characteristics of the AMI patients**

| **Characteristic** |  |
| --- | --- |
| Ages（years） | 64.30 (59.00-69.00) |
| Male/Female | 48/20 |
| Hypertension with well controlled（%） | 45/68 |
| Diabetes mellitus with well controlled（%） | 17/68 |
| Old myocardial infarction | 0/68 |
| Prior PCI | 0/68 |
| Prior CABG | 0/68 |
| Atrial fibrillation | 0/68 |
| Hyperlipemia with well controlled（%） | 34/68 |
| Chronic Heart failure | 0/68 |
| **Laboratory and auxiliary examinations** |  |
| Systolic blood pressure (mmHg) | 129.7 (116.0-141.5) |
| Diastolic blood pressure(mmHg) | 75.68(66.00-84.50) |
| Ejection fraction (%) | 53.14 (46.50-60.00) |
| cTNT (ng/ml) | 2.645 (0.9020-5.615) |
| CK-MB(U/L) | 132.60 (27.50-127.0) |
| NT-proBNP (pg/mL) | 588.0 (131.7-1809) |
| ALT (U/L) | 42.90 (18.00-48.00) |
| hrCRP | 17.73 (2.20- 21.50) |
| White blood cell count (×10^9^/L) | 10.42 (7.413-11.93) |
| Hemoglobin (g/L) | 131.6 (119.0-144.3) |
| Total cholesterol (mmol/L) | 4.404(3.693-5.150) |
| Triglyceride (mmol/L) | 1.440 (0.7225- 1.748) |
| High-density lipoprotein (mmol/L) | 1.1160 (0.9250-1.275) |
| Low-density lipoprotein (mmol/L) | 2.636 (2.040-3.090) |
| **Medication on admission** |  |
| Diuretics, n (%) | 9/68 |
| β-blocker, n (%) | 23/68 |
| ACEIs or ARBs, n (%) | 23/68 |
| CCBs, n (%) | 19/68 |
| Aspirin, n (%) | 30/68 |
| Statin | 34/68 |

Data are expressed as Mean ± SD, n (%) or. Median [ interquartile range].

PCI：Percutaneous Coronary Intervention; CABG; Coronary Artery Bypass Grafting; ACEI: Angiotensin-Converting Enzyme Inhibitors; ARB: Angiotensin Receptor Blockers; CCB: Calcium Channel Blockers.

|  | sham | | I/R | |
| --- | --- | --- | --- | --- |
|  | Vehicle | rbWnt2 | Vehicle | rbWnt2 |
| LVAWs (mm) | 1.274±0.1062 | 1.416±0.2407 | 1.246±0.1177 | 1.272±0.1020 |
| LVAWd  (mm) | 0.7750±0.1046 | 0.8965±0.1134 | 0.7490±0.06358 | 0.7817±0.09817 |
| LVIDs  (mm) | 2.380±0.1854 | 2.440±0.1038 | 4.016±0.1392^**^ | 2.993±0.1237^##^ |
| LVIDd  (mm) | 3.362±0.06575 | 3.327±0.1436 | 4.744±0.1122^*^ | 4.210±0.06972^#^ |
| Temp  (°C) | 35.98±0.3594 | 36.18±0.4924 | 35.84±0.3395 | 36.02±0.3193 |

**Table S2. The parameters relative to echocardiography analysis in sham or I/R mice with or without Wnt2 treatment.**

LVAWs： Left Ventricular Anterior Wall End-Systolic thickness; LVAWd： Left Ventricular Anterior Wall End-Diastolic thickness; LVIDs： Left Ventricular Internal Dimension End-Systolic; LVIDdLeft Ventricular Internal Dimension End-Diastolic. Data are expressed as Mean ± SD, *p <0.001; **p < 0.0008 vs sham Vehicle group; ^#^P<0.001;^##^P <0.0001 vs I/R Vehicle group. n=7-10

**Table S3. Analysis of heart rate (HR) in mice with or without rb Wnt2 treatment during I/R.**

|  | Pre-Ischemia | Ischemia | Just after Reperfusion | Reperfusion 24h |
| --- | --- | --- | --- | --- |
| Vehicle | 554.4±53.18 | 465.7±29.6 | 551.0±28.67 | 436.7±35.69 |
| rbWnt2 | 564.8±46.62 | 517.1±64.42 | 575.5±18.91 | 444.2±44.27 |
| P value | 0.7505 | 0.0790 | 0.2093 | 0.7613 |

Data are expressed as Mean ± SD, n=7-9.

**
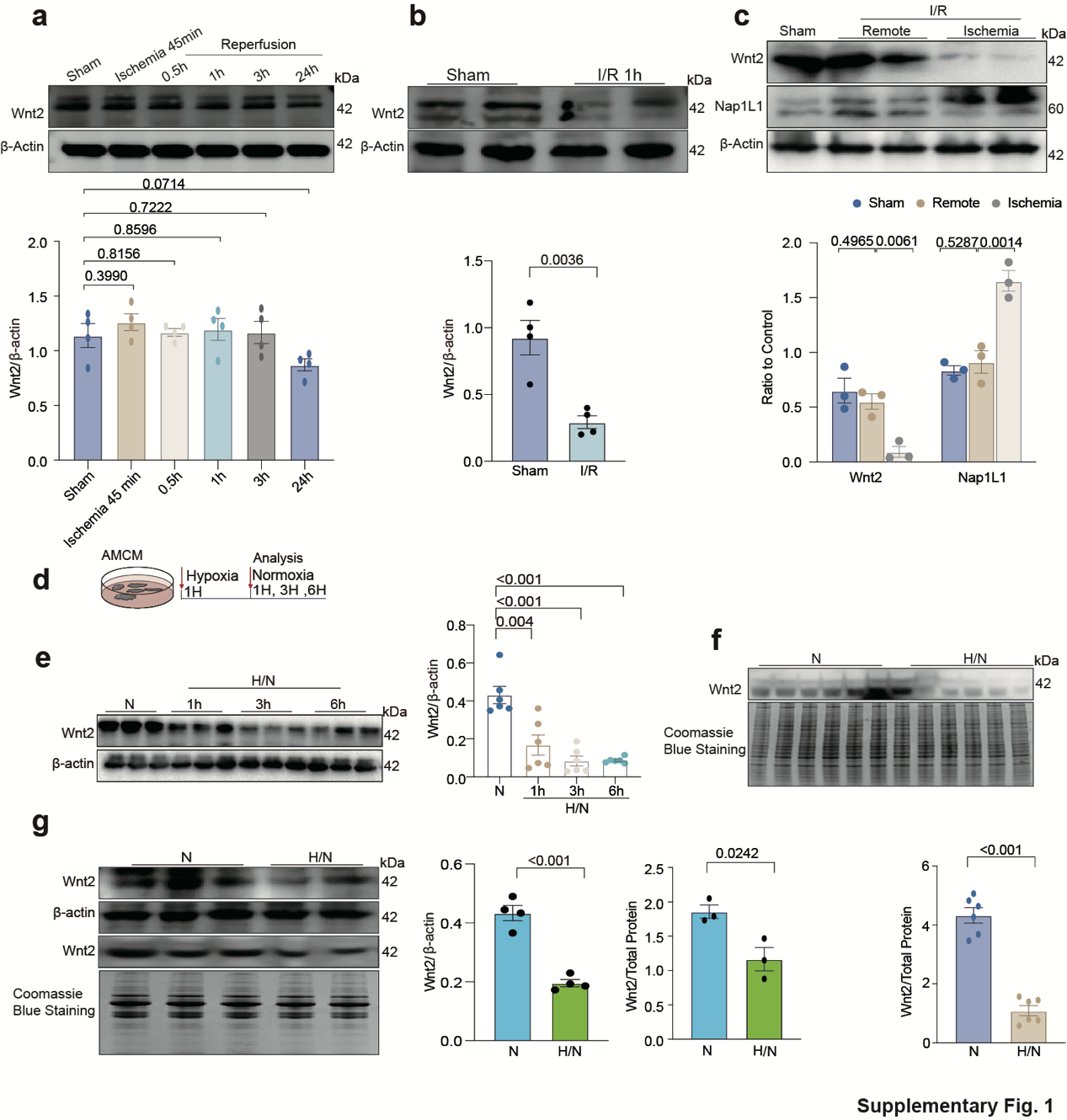
**

**Supplementary Fig.1 Wnt2 decreased during cardiac I/R injury.**

**a** Western blot analysis of Wnt2 in the ischemic area of mice with I/R injury at various time points starting from ischemia onset. n = 4/group. **b** Western blot analysis of Wnt2 in isolated cardiomyocytes from mice subjected to I/R injury. n = 4/group. **c** Western blot analysis of Wnt2 in both ischemic and non-ischemic (remote) areas. n = 3/group. **d** Experimental design: Isolated cardiomyocytes were treated with rbWnt2 or PBS prior to hypoxia (1 h)/normoxia. **e** Western blot analysis of Wnt2 in isolated cardiomyocytes of adult mice (AMCMs) undergoing hypoxia 1h /normoxia (H/N) at different normoxia time points. N=6/group. **f** Western blot analysis of Wnt2 in conditional medium (CM) from AMCMs undergoing hypoxia/normoxia (H/N) or normaoxia (N). n=6/group. **g** Western blot analysis of Wnt2 in cultured cardiac fibroblasts and their conditional medium undergoing hypoxia/normoxia (H/N) or normaoxia (N). n=3-4/group. All the data were expressed by mean±SEM.


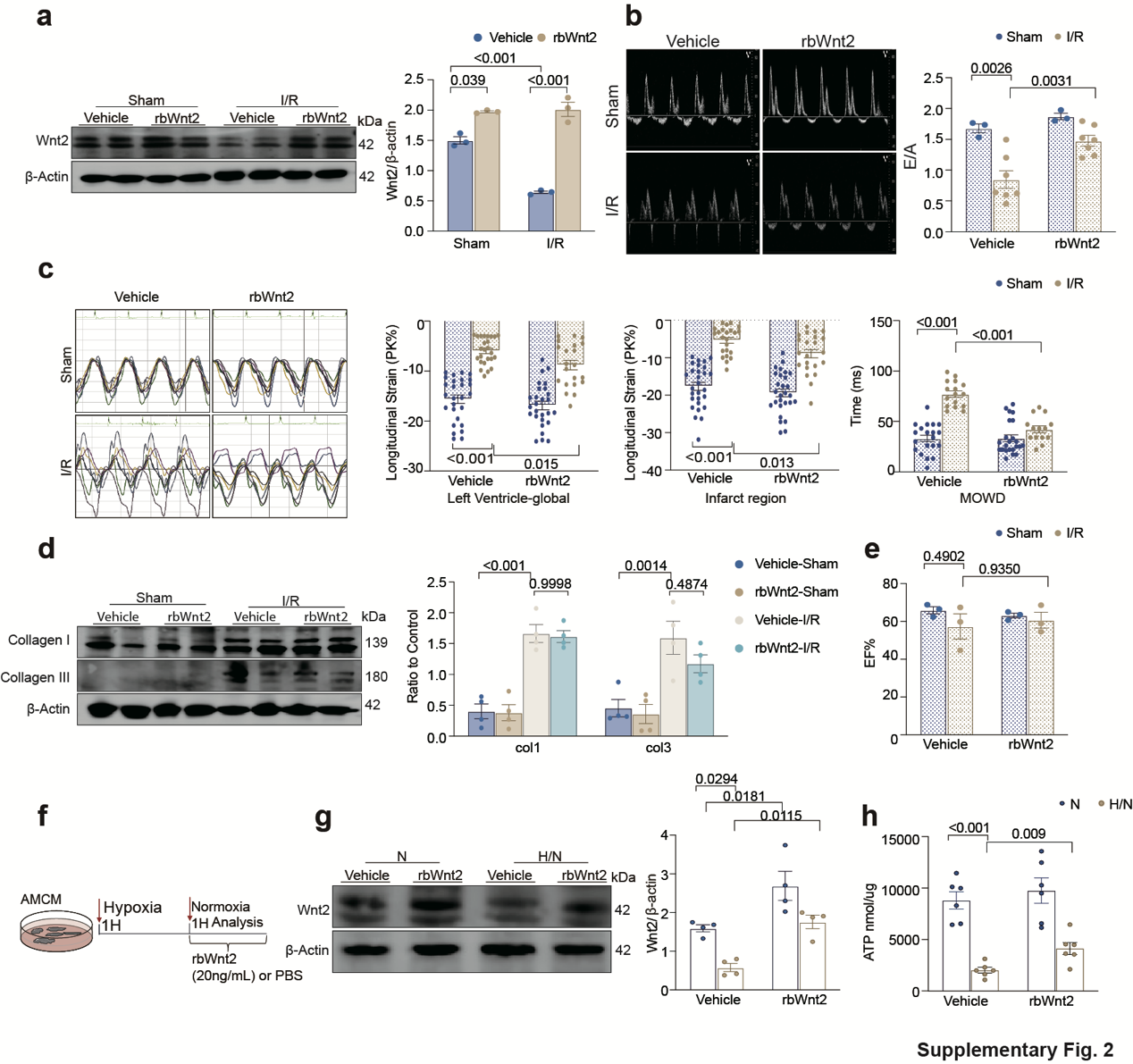


**Supplementary Fig.2 The administration of rbWnt2 significantly mitigates cardiac I/R injury.**

**a** Western blot analysis of Wnt2 in I/R or sham hearts from mice pretreated with rbWnt2 or PBS. n=3/group. **b** Representative pulsed-wave Doppler and quantitative analysis of left ventricular diastolic function (E/A ratio) post-I/R. Mice were pretreated with rbWnt2 or PBS. n= 3-7/group. **c** Longitudinal strain analysis in mice. Left lane: Representative images of longitudinal strain curves. Colored lines represent 6 standard myocardial regions; Right lane: Quantitative analysis of longitudinal strain both global and infarcted area and maximal opposite wall delay in time (MOWD). n=21-27/group. **d** Western blot analysis of Collagen I and Collagen III in I/R or sham hearts from mice pretreated with rbWnt2 or PBS. n=4/group. **e** Left ventricular ejection fraction (LVEF) at two weeks post-I/R. Mice were pretreated with rbWnt2 or PBS. N=3/group. **f** Experimental design: Isolated cardiomyocytes were treated with rbWnt2 or PBS prior to hypoxia (1 h)/normoxia (1 h). **g** Western blot analysis of Wnt2 in cultured adult mouse cardiomyocytes (AMCMs) after H/N pretreated with rbWnt2 or PBS (as control) n=4/group. **h** ATP levels in cultured adult mouse cardiomyocytes (AMCMs) after H/N pretreated with rbWnt2 or PBS (as control) n=6/group. All the data were expressed by mean±SEM.


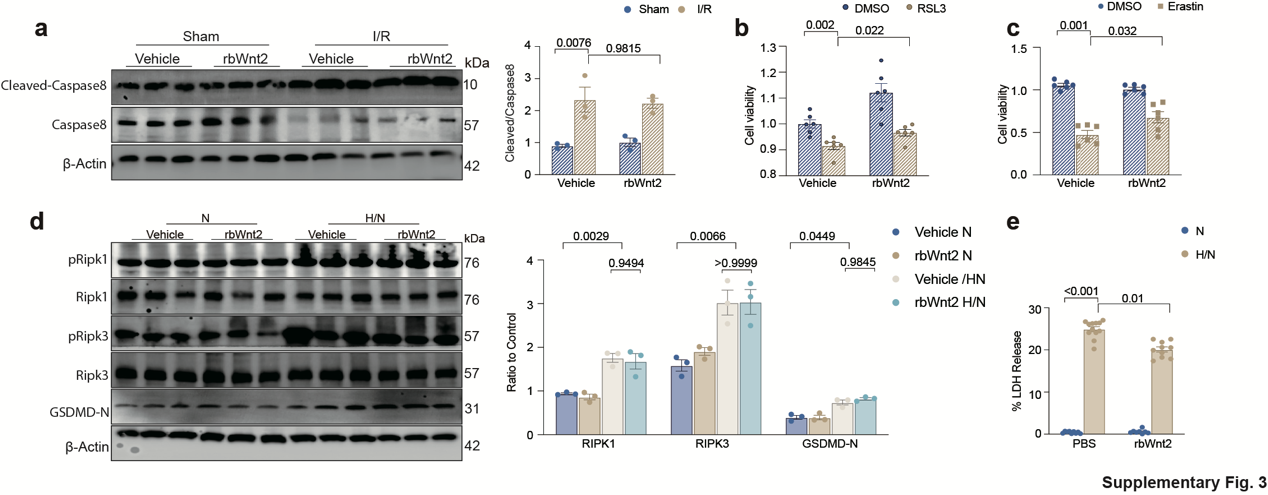


**Supplementary Fig. 3 The effects of rbWnt2 intervention on cardiomyocyte death during myocardial I/R injury.**

**a** Immunoblot analysis of extrinsic apoptosis related protein Cleaved-Caspase8 and Caspase8 in cultured adult mouse cardiomyocytes (AMCMs) under H/N or normoxia (N) treated with rbWnt2 or PBS. n=3/group. **b** and **c** Cell viability of AMCMs treated with 10μM RSL3 (b), or 20μM Erastin (c). DMSO was used as a control. n=6/group. **d** Immunoblot analysis of necroptosis and pyroptosis related proteins (Ripk1, 3, p-Ripk1,3 and GSDMD-N) in cultured AMCMs under H/N or N treated with rbWnt2 or PBS. n=3/group. **e** LDH release from AMCMs under H/N or N treated with rbWnt2 or PBS. n=9-12/group. All data are expressed as mean ± SEM.


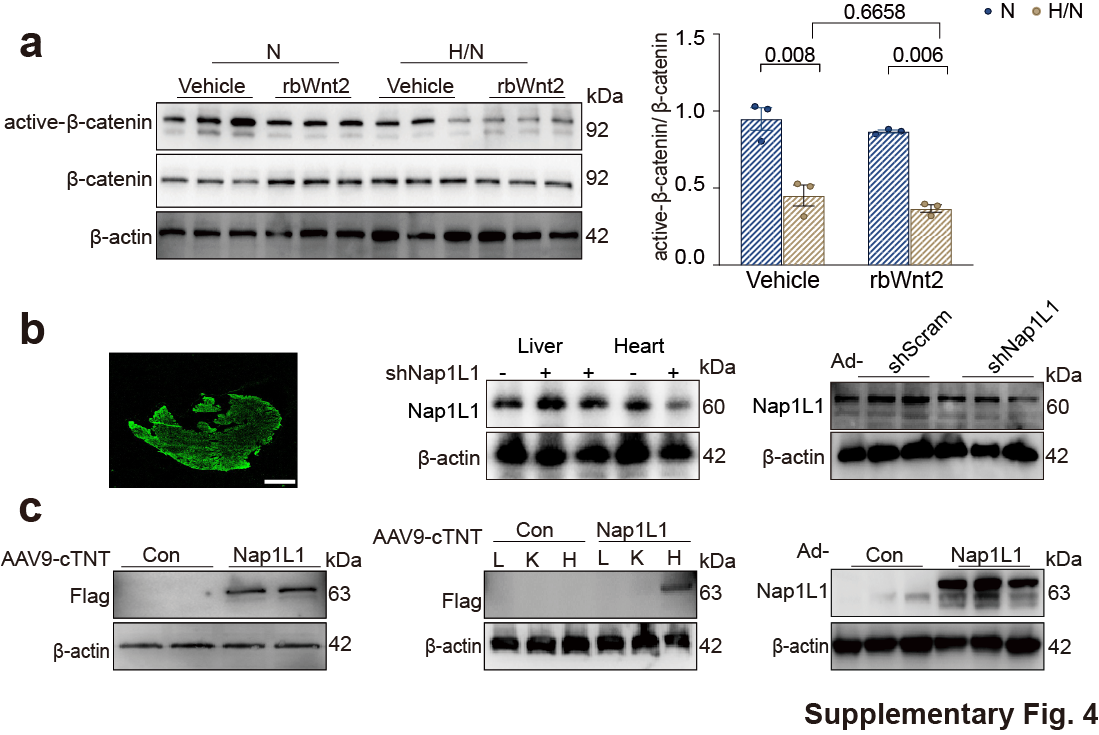


**Supplementary Fig.4 RbWnt2 promotes ROS scavenging genes transcription independently of canonical Wnt/β-catenin signaling.**

**a** Western blot analysis of active β-catenin and total β-catenin in cardiomyocytes treated with rbWnt2 or PBS (vehicle) and subjected to H/N. n = 3/group. **b** Left panel: Representative fluorescence image of cardiac frozen sections from mice injected with AAV9-cTNT-ZsGreen-shNap1L1 by the tail vein. Middle panel: Western blot analysis of Nap1L1 expression in heart and liver from mice injected with AAV9-cTNT-ZsGreen-shNap1L1 and AAV9-cTNT-ZsGreen-NC in the tail vein. Right panel: Western blot analysis of Nap1L1 expression in cultured adult murine cardiomyocytes (AMCMs) transfected with adenovirus shRNA-Nap1L1 (shNap1L1) and shRNA-shscramble (Ad-shScramble). Scale bar, 200μm. **c** Left panels: Western blot analysis of Flag expression in heart (H), kidney (K), and liver (L) tissues from mice injected with AAV9-cTNT-Nap1L1 via the tail vein. Right panel: Western blot analysis of Nap1L1 expression in cultured adult murine cardiomyocytes (AMCMs) transfected with adenovirus Nap1L1 (Ad-Nap1L1) or control (Ad-control). All data are expressed as mean ± SEM.

**
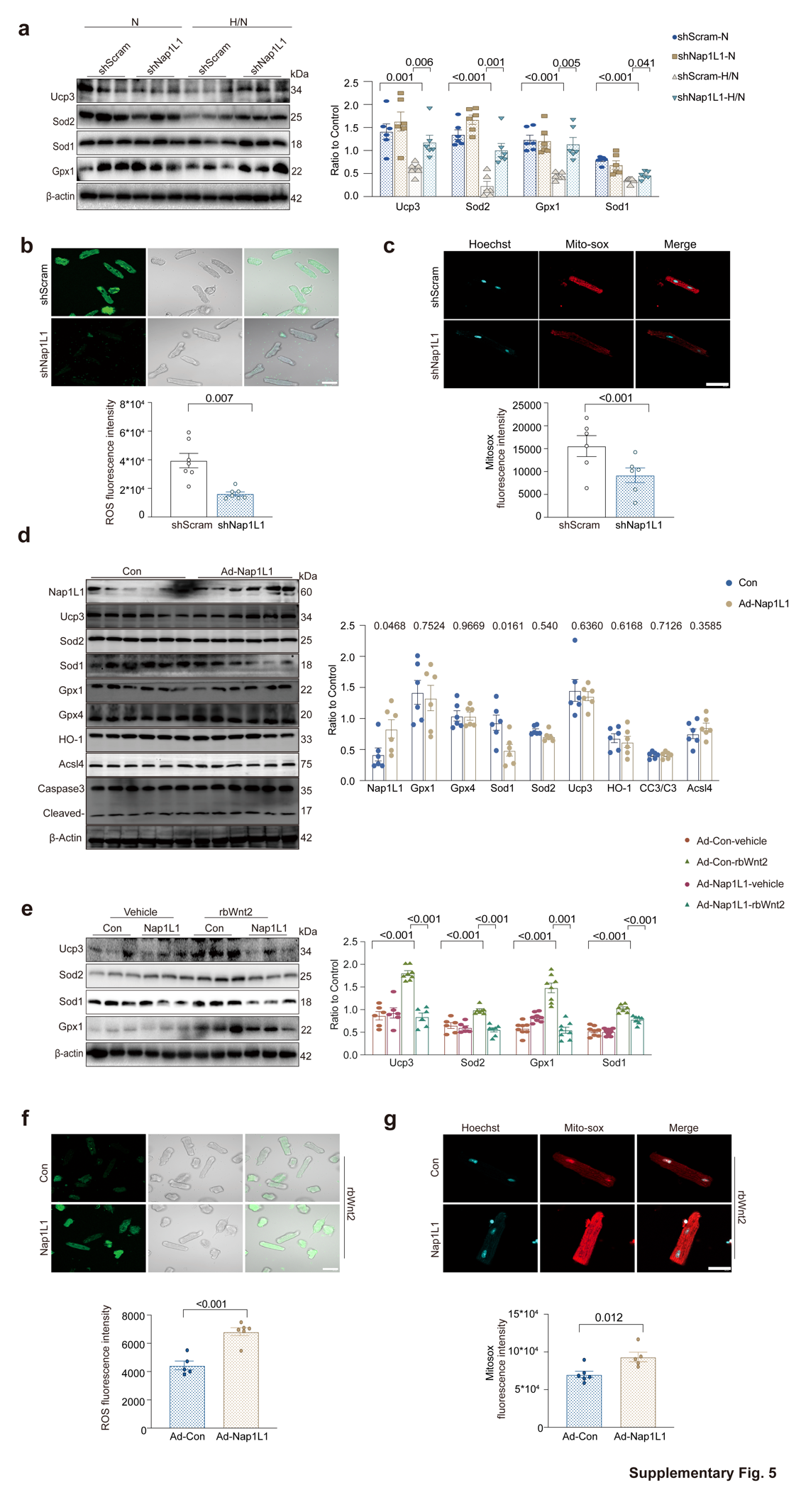
**

**Supplementary Fig.5 RbWnt2 promotes ROS scavenging genes transcription through down-regulation of Nap1L1.**

**a** Western blot analysis of Ucp3, Sod2, Sod1 and Gpx1 in AMCMs transfected with adenovirus shRNA-Nap1L1(shNap1L1) or shRNA-scramble and then subjected to H/N.n=6/ group. **b** Representative DCFH-DA staining of AMCMs transfected with adenovirus shRNA-Nap1L1(shNap1L1) or shRNA-scramble and then subjected to hypoxia/normoxia (H/N). Scale bar, 50μm. n=7/group. **c** Mito-sox staining of AMCMs treated with adenovirus shRNA-Nap1L1(shNap1L1) or shRNA-scramble under H/N environment. Scale bar, 50μm. n=6/group. **d** Western blot analysis of Ucp3, Sod2, Sod1,HO-1,Acsl4, total and cleaved Caspase3 and Gpx1 levels in AMCMs transfected with adenovirus Nap1L1 under basal condition. n=6/group. **e** Western blot analysis of Ucp3, Sod2, Sod1 and Gpx1 levels in H/N injured AMCMs transfected with adenovirus Nap1L1. n=6-8/group. **f** Representative DCFH-DA staining of AMCMs treated with adenovirus Nap1L1 under H/N environment. Scale bar, 50 μm, n=5-6/group. **g** Mito-sox staining of AMCMs in groups of (**f**). Scale bar, 50μm. n=5-6/group. All data are expressed as mean ± SEM.


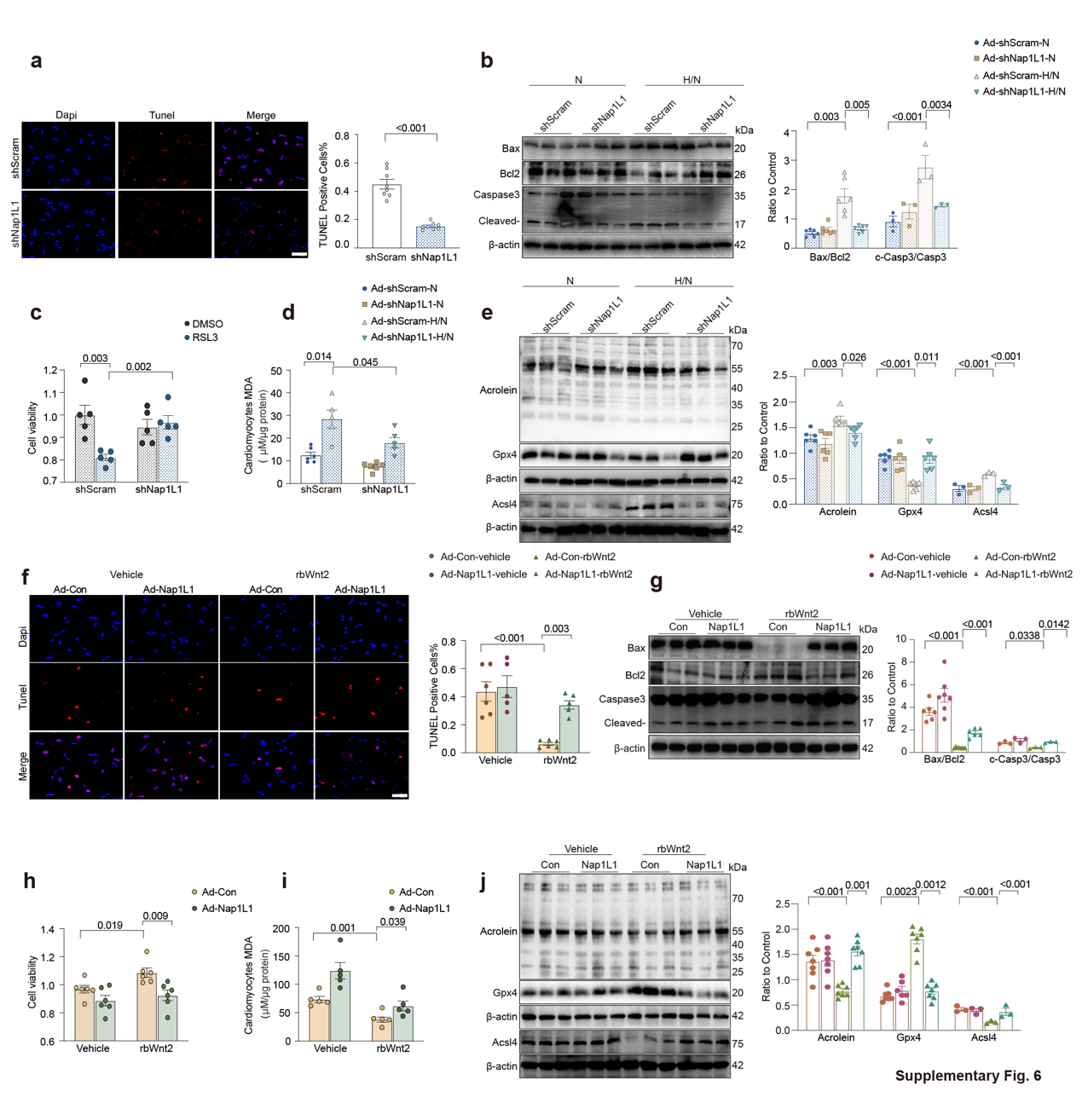


**Supplementary Fig.6 Wnt2 downregulation of Nap1L1 suppresses cardiac apoptosis and ferroptosis in I/R hearts.**

**a** TUNEL staining analysis of AMCMs transfected with adenovirus shRNA-Nap1L1 (shNap1L1) or shRNA-scramble (shScramble) and subjected to H/N. Scale bar: 50 μm. n= 8/group. **b** Western blot analysis of Bax, Bcl2 and cleaved-caspase3,caspase 3 levels in AMCMs transfected with adenovirus shRNA-Nap1L1(shNap1L1) or shRNA-scramble and then subjected to hypoxia/normoxia (H/N). n=3-6/group. **c** Analysis of cell viability of adult mouse cardiomyocytes (AMCMs). AMCMs were transfected with adenovirus shRNA-Nap1L1 (ShNap1L1) or shRNA-scramble and treated with 10μM RSL3 orDMSO (as control) for 24h. n=5/group. **d** MDA levels in AMCMs under H/N with or without Nap1L1 knockdown. n= 5–6/group. **e** Western blot analysis of Acrolein, Gpx4 and Acsl4 expression in AMCMs under H/N with or without Nap1L1 knockdown. n=3-6/group. **f** TUNEL staining analysis of AMCMs transfected with adenovirus-Nap1L1 (Ad-Nap1L1) or control (Ad-control) and subjected to H/N. Scale bar: 50μm. n= 5–6/group. **g** Western blot analysis of apoptosis related protein Bax, Bcl2, cleaved-caspase3, Caspase3 levels in AMCMs pre-treated with Ad-Nap1L1 or Ad-control and rbWnt2 or vehicle. n=3-7/group. **h** Analysis of cell viability of AMCMs transfected with adenovirus Nap1L1(Ad-Nap1L1) or control (Ad-control) and treated with10μM RSL3 or DMSO (as control) for 24h in. n=6/group. **i** MDA levels in H/N-treated AMCMs with or without Nap1L1 overexpression. n= 5/group. **j** Western blot of Acrolein and Gpx4 expression in H/N-treated AMCMs with or without Nap1L1 overexpression. n= 3-7/group. All data are expressed as mean ± SEM.


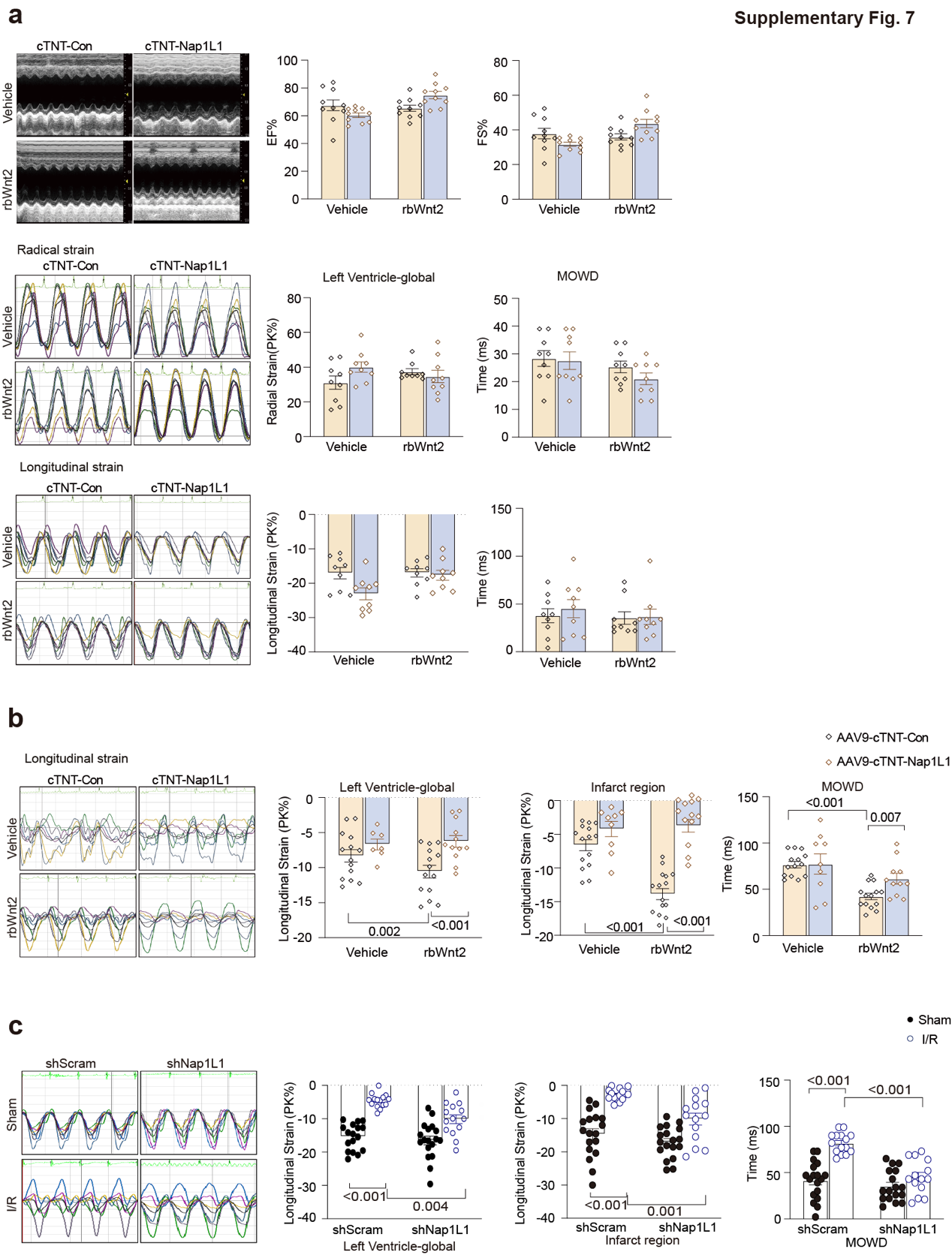


**Supplementary Fig.7 Regulation of Nap1L1 is critical for cardiac protection of Wnt2 following I/R injury.**

**a** Upper panel: Representative M-mode echocardiograms and quantitative analysis of left ventricular EF and FS in mice pre-treated with AAV9-cTNT- Nap1L1 or AAV9-cTNT- control and rbWnt2 or vehicle at basic line. Middle and Lower panel: Radial and longitudinal strain analysis global and MOWD in mice injected with AAV9-cTNT-Nap1L1 in the tail vein at basic line obtained from Vevo Strain analysis software. n=9-14/group. **b** Longitudinal strain analysis in mice pre-treated with AAV9-cTNT- Nap1L1 or AAV9-cTNT-control and rbWnt2 or vehicle subjected to I/R injury. Left panel: Representative images of longitudinal strain curves. Colored lines represent 6 standard myocardial regions; Right panel: Quantitative analysis of longitudinal strain both global and infarct area and MOWD. N=7-15/group. **c** Longitudinal strain analysis in mice injected with AAV9-cTNT-ZsGreen-shNap1L1 and AAV9-cTNT-ZsGreen-NC in the tail vein. Left panel: Representative images of longitudinal strain curves. Colored lines represent 6 standard myocardial regions; Right panel: Quantitative analysis of longitudinal strain (global, infarct area, and MOWD). n=14-18/group. All data are expressed as mean ± SEM.

**
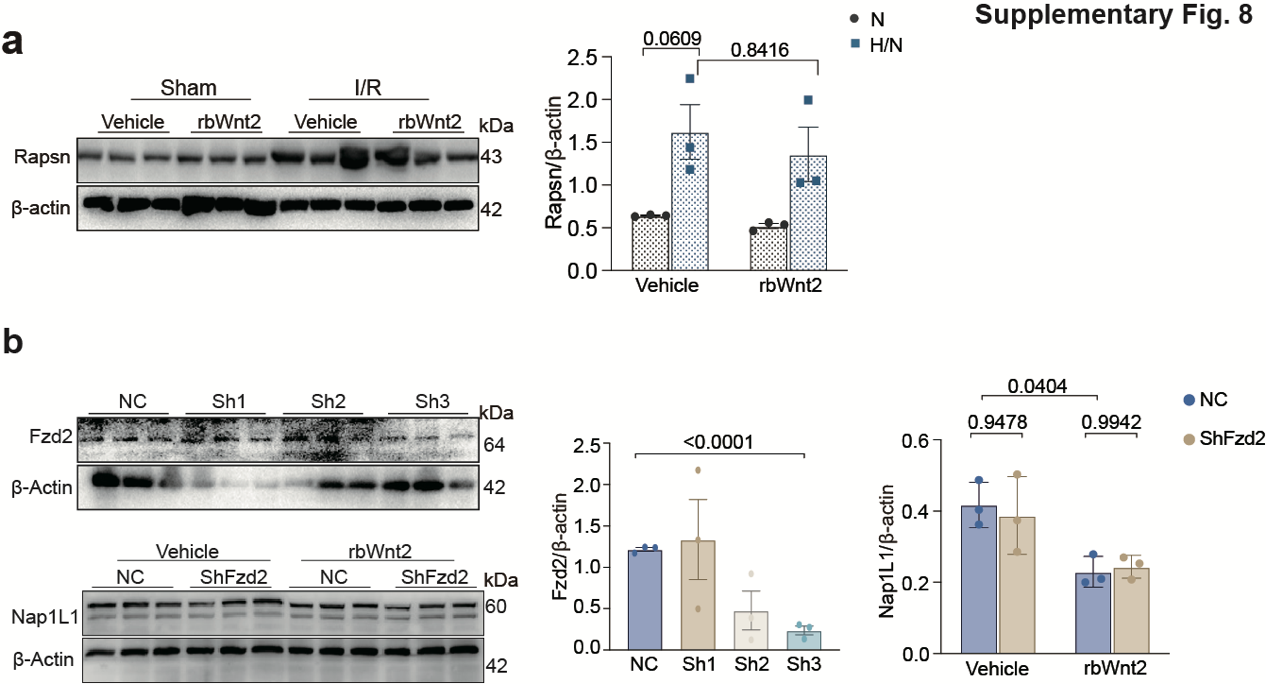
**

**Supplementary Fig.8** **rbWnt2 acts on Lrp6 to promote Nap1L1 degradation by Trim11 in response to I/R.**

**a** Rapsn expression and quantification in cardiomyocytes treated with rbWnt2 or PBS (as control) cultured under H/N environment. n=3/group. **b** Upper panel: Western blot analysis of Fzd2 expression in cardiomyocytes transfected with shFzd2 or NC. Lower panel: Western blot analysis of Nap1L1 expression in AMCMs transfected with shFzd2 or NC, treated with PBS or rbWnt2 under H/N. n= 3/group. All data are expressed as the mean ± SEM.
